# A rationally designed rhodamine-based fluorescent probe for molecular imaging of peroxynitrite in live cells and tissues†
†Electronic supplementary information (ESI) available: Detailed experimental procedures for the organic synthesis, photophysical characterization and supplementary imaging figures. See DOI: 10.1039/c6sc00012f


**DOI:** 10.1039/c6sc00012f

**Published:** 2016-04-26

**Authors:** Tao Peng, Xingmiao Chen, Lei Gao, Ting Zhang, Wei Wang, Jiangang Shen, Dan Yang

**Affiliations:** a Morningside Laboratory for Chemical Biology , Department of Chemistry , The University of Hong Kong , Pokfulam Road , Hong Kong , P. R. China . Email: yangdan@hku.hk; b School of Chinese Medicine , The University of Hong Kong , Pokfulam Road , Hong Kong , P. R. China

## Abstract

Herein, we present the rational design, synthesis, characterization, and biological applications of a new rhodamine-based fluorescent probe, HKYellow, for the detection and molecular imaging of peroxynitrite, an important highly reactive oxidant involved in a variety of physiological and pathological processes. HKYellow was rationally designed on the basis of the efficient fluorescence quenching effect of the *N*-phenyl group to the rhodamine core and a peroxynitrite-triggered *N*-dearylation reaction to achieve a sensitive and selective fluorescence turn-on response toward peroxynitrite in chemical and biological levels. This probe has been thoroughly evaluated for the robust imaging of peroxynitrite in live cells and tissues. By utilizing HKYellow, we provide the first visual evidence that peroxynitrite is generated in mouse liver tissues under an acute alcohol binge or ischemic–reperfusion condition. This probe should be a powerful molecular imaging tool for interrogating the complex biological roles of peroxynitrite under various biological settings.

## Abstract 2

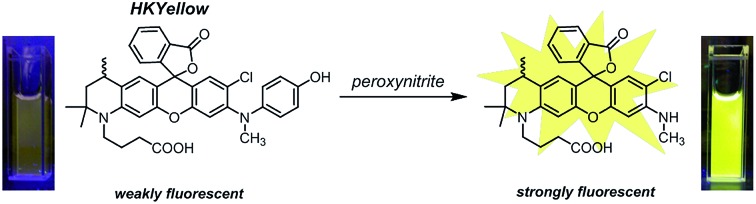
A novel fluorescent reporter HKYellow was rationally designed for the robust visualization of peroxynitrite production in live cells and tissues.

## Introduction

Reactive oxygen species (ROS) and reactive nitrogen species (RNS) refer to oxygen-containing and nitrogen-containing molecules with a high reactivity to biomolecules, respectively. They are involved in a wide variety of biological events, including oxidative damage, cellular signalling, and immune response.^1^,^2^ Among various ROS and RNS, peroxynitrite (ONOO^–^) is often included as an ROS because of its strong oxidizing ability,^3^ although it is a nitrogen-containing species. Peroxynitrite is formed from the immediate *in vivo* reaction of superoxide (O_2_˙^–^) and nitric oxide (˙NO). As a strong oxidant, peroxynitrite interacts with a panel of biomolecules, including lipids, DNA, and proteins, *via* direct oxidation or indirect secondary radical-mediated reactions and causes serious irreversible damage to them.^4^ Emerging data have shown that peroxynitrite is responsible for most of the cytotoxicity that was traditionally attributed to its precursors O_2_˙^–^ and ˙NO. As a result, peroxynitrite generation is thought to represent an important pathogenic mechanism in a series of diseases such as neurodegeneration, ischemia–reperfusion injury, inflammation, vascular diseases, circulatory shock, and cancer.^5^,^6^

Most of the current studies on peroxynitrite rely on indirect measurements and its molecular footprints, *e.g.*, tyrosine nitration, which inevitably results in doubts and debates about its biological relevance.^7^ Peroxynitrite specific fluorescent probes allowing for *in situ* detection, spatial and temporal imaging should be powerful tools for directly monitoring peroxynitrite generation and interrogating its biological roles.^7^ In this regard, a panel of fluorescent probes for peroxynitrite detection has been developed over the past decade.^8^–^26^ However, most of these probes are not sensitive and selective enough to unambiguously detect endogenous peroxynitrite at low concentrations. Moreover, only a few are thoroughly characterized and validated at biochemical and cellular levels for practical applications.

Previously, we reported a series of green fluorescent probes for peroxynitrite detection and imaging.^14^–^17^,^26^ We were further motivated to develop probes operating in the yellow, red, and even far-red regions, as such long-wavelength probes are well-documented to be more desirable for biological applications in terms of their low photo-induced damage, low intracellular autofluorescence, deeper tissue penetration, and compatibility for multicolor imaging.^27^,^28^ Herein, we report the rational design and characterization of HKYellow, a small-molecule yellow fluorescence turn-on probe used for the sensitive and selective detection of peroxynitrite in chemical systems and live biological samples.

## Results and discussion

### Design and synthesis of HKYellow

**Fig. 1:**
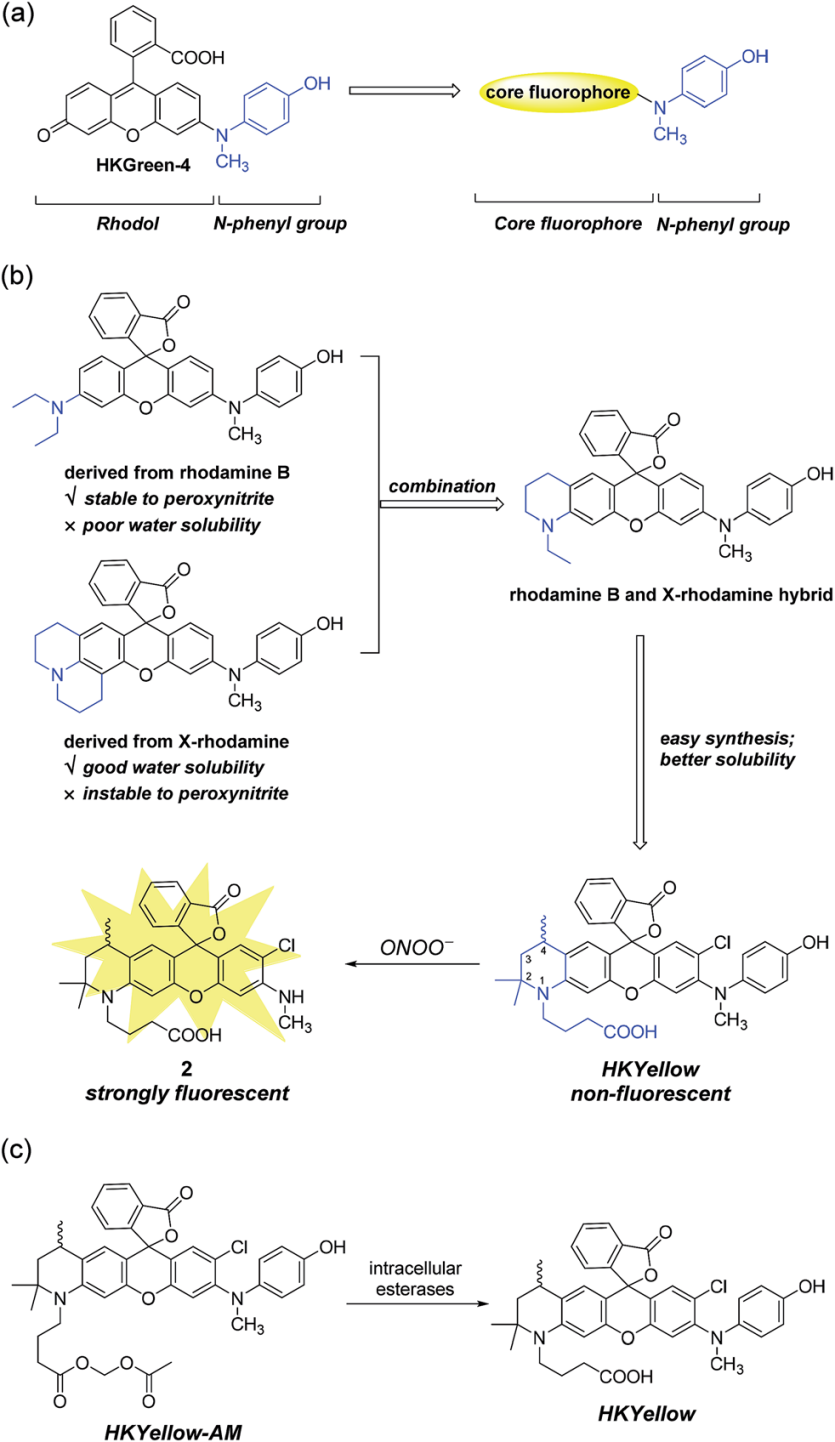
Rational design of HKYellow used for the detection of peroxynitrite. (a) Generalization of HKGreen-4 for designing new peroxynitrite probes with varying excitation and emission colors. (b) Structural combination of rhodamine B and X-rhodamine and incorporation of a carboxylate group leading to the new rhodamine scaffold used for constructing HKYellow, which is virtually non-fluorescent yet reacts with peroxynitrite to yield a strongly fluorescent product **2**. (c) The structure of the cell membrane permeable HKYellow-AM and its conversion to HKYellow by intracellular esterases.

To design probes with expanded excitation and emission colors, we recalled the structure of our latest peroxynitrite probe HKGreen-4 (Fig. 1a), which was developed by utilizing the *N*-dearylation reaction of *N*-(4-hydroxylphenyl)-rhodol exerted by peroxynitrite.^26^ It was speculated that the probe can be structurally and functionally divided into two parts, *i.e.*, the rhodol part and the *N*-phenyl group (Fig. 1a). The rhodol part is the source of fluorescence, determining the photophysical properties of the probes, such as fluorescence colors, pH dependence, and photostability. On the other hand, the *N*-phenyl group quenches the fluorescence of the rhodol fluorophore and reacts with peroxynitrite, thus regulating the sensitivity of the probe to peroxynitrite. We envisioned that the *N*-phenyl group could also quench the fluorescence of other xanthene fluorophores and therefore, substitution of the rhodol fluorophore with other long-wavelength xanthene fluorophores would provide probes with distinct excitation and emission colors (Fig. 1a). Moreover, the electron-rich peroxynitrite-reactive *N*-phenyl group should be retained to ensure the probes sensitivity and selectivity to peroxynitrite (Fig. 1a).

Initially, we chose rhodamine as the core fluorophore to demonstrate this concept because rhodamine dyes exhibit tunable fluorescence, great photostability, and pH-insensitivity.^28^ However, the probes derived from commonly used rhodamine dyes, such as rhodamine B and X-rhodamine (Fig. 1b), were unfortunately not suitable for peroxynitrite detection, mainly because of either their insufficient stability to strongly oxidative peroxynitrite or poor water solubility. For instance, the julolidine moiety of X-rhodamine, in spite of increasing water solubility, is not stable toward peroxynitrite due to the exposed lone electron pair of nitrogen, which is prone to peroxynitrite attack. On the contrary, the *N*,*N*-diethylaniline moiety from rhodamine B is stable to peroxynitrite, probably owing to steric protection of the nitrogen lone electron pair by ethyl groups. However, the derived probe suffers from poor solubility in an aqueous solution, precluding its use for peroxynitrite detection.^29^ We reasoned that the structural combination of the julolidine moiety of X-rhodamine and the *N*,*N*-diethylaniline moiety of rhodamine B, leading to an *N*-ethyl-2-methyl-tetrahydroquinoline moiety (Fig. 1b) would overcome the stability and solubility problems by inheriting advantages and counteracting the drawbacks from the parental moieties. For ease of synthesis and better water solubility, an analogous *N*-(3-carboxypropyl)-2,2,4-trimethyl-tetrahydroquinoline moiety was incorporated into the probe (Fig. 1b). We anticipated that the rigid six-member-ring tetrahydroquinoline moiety would benefit from a bright and red-shifted fluorescence^28^,^30^ and the two α methyl groups near the nitrogen atom would provide a steric shield to the nitrogen lone-pair electrons, thus blocking the attack from peroxynitrite. Moreover, the *N*-carboxypropyl group was introduced to further improve the water solubility of the probe. Thus, we synthesized this rationally designed rhodamine-based peroxynitrite probe, named HKYellow (Fig. 1b) from simple building blocks (for synthetic details, see Scheme S1 in the ESI†).

### Reactivity and selectivity of HKYellow for peroxynitrite

**Fig. 2:**
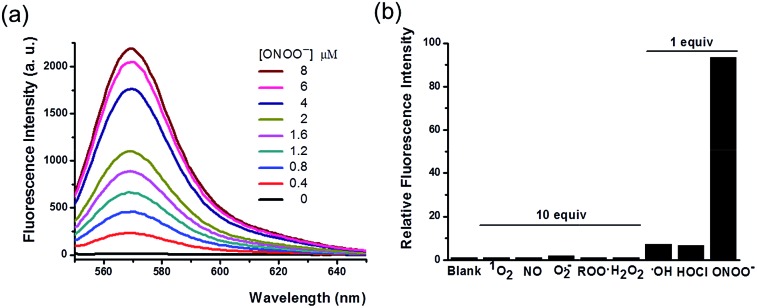
Photophysical characterization of HKYellow for detecting peroxynitrite. (a) Fluorescence responses of 2 μM HKYellow to different amounts of ONOO^–^. (b) The relative fluorescence intensity (*λ*_em_ = 570 nm) of 2 μM HKYellow toward various ROS and RNS. HKYellow was dissolved in 0.1 M phosphate buffer at pH 7.4 and excited at 545 nm. ROS and RNS were added into the probe solution slowly with vigorous stirring. The reactions were then carried out for 30 min at room temperature in the dark before the fluorescence intensity was measured.

With the probe in hand, we tested its photophysical properties and reactivity towards peroxynitrite in aqueous solutions buffered at physiological pH (0.1 M phosphate buffer, pH 7.4). HKYellow is readily soluble in water, exhibiting a strong absorption peak at 560 nm (*ε* = 60 000 M^–1^ cm^–1^, Fig. S1 in the ESI†). To our delight, HKYellow was virtually non-fluorescent in an aqueous solution (*Φ* = 0.034; Fig. 2a), suggesting that the *N*-phenyl group substituted on one nitrogen of rhodamine was sufficient to quench its fluorescence, probably through torsional relaxation and/or excited-state electron transfer.^31^ The addition of peroxynitrite to a solution of HKYellow (2 μM in phosphate buffer, pH 7.4) resulted in a robust and immediate fluorescence turn-on response with the maximal emission at 570 nm in a dose-dependent manner (Fig. 2a). In particular, a 93-fold fluorescence increase of HKYellow was detected with an apparent linear relationship between the fluorescence intensity and peroxynitrite concentration (Fig. S2 in ESI†). The fluorescence increase was completed in less than 2 seconds after the addition of peroxynitrite. Moreover, the fluorescent product was found to be stable to photoirradiation over one hour (Fig. S3 in the ESI†). Notably, HKYellow has fluorescence excitation and emission maxima at 545 nm and 570 nm, respectively, almost the same as those of the widely used tetramethylrhodamine and perfectly matching the red filter setting in epifluorescence microscopy and the He–Ne 543 nm excitation laser commonly used in confocal microscopy.

The effective pH range of HKYellow for detecting peroxynitrite was determined to be from 6 to 9.5, a broad range with biological relevance (Fig. S4 in the ESI†). The addition of DMSO, a well-known effective ˙OH scavenger,^32^ into the probe solution at various concentrations (up to 2% v/v) hardly affected the fluorescence response of HKYellow to peroxynitrite (Fig. S5 in the ESI†). To investigate the reaction product of HKYellow with peroxynitrite, an expected *N*-dearylation product **2** was synthesized and characterized (*λ*_ex_ = 543 nm, *λ*_em_ = 570 nm, *Φ* = 0.53; see Fig. S1 in the ESI†). Both HPLC and LC-MS analyses of the reaction mixture confirmed the generation of the *N*-dearylation product **2** as the fluorescence source (Fig. S6 in the ESI†).

The reactivity of HKYellow toward a panel of ROS and RNS was also assessed to evaluate the selectivity of the probe for detecting peroxynitrite (Fig. 2b). As expected, most of biologically relevant ROS and RNS, including H_2_O_2_, ^1^O_2_, ˙NO, O_2_˙^–^, and ROO˙, even presented in 10 equiv., failed to induce an increase in fluorescence. Moreover, potential interfering highly reactive oxygen species (hROS), such as HOCl and ˙OH,^33^,^34^ only triggered fluorescence increases of HKYellow to a significantly lower extent when compared to peroxynitrite (Fig. 2b).

### Evaluation of HKYellow for imaging peroxynitrite in live cells

**Fig. 3:**
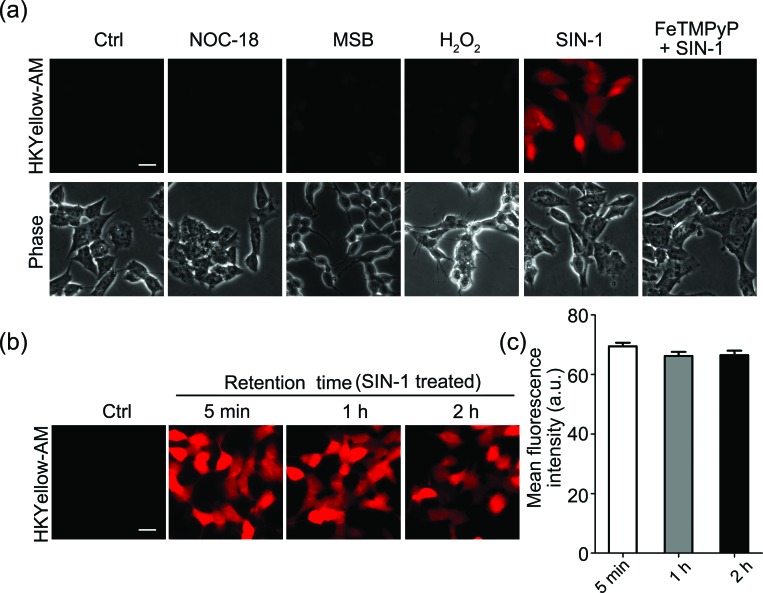
Characterization of HKYellow-AM for imaging peroxynitrite in live cells. (a) Validation of HKYellow selectivity with exogenous ROS/RNS donors in SH-SY5Y human neuroblastoma cells. The cells were firstly incubated with HKYellow-AM (10 μM) and then treated with H_2_O_2_ (100 μM) or the indicated ROS/RNS donors for 1 h, followed by fluorescence imaging. NOC-18 (1 mM), MSB (200 μM), and SIN-1 (100 μM) were used to produce ˙NO, O_2_˙^–^, and ONOO^–^, respectively. FeTMPyP (50 μM) was used as an ONOO^–^ decomposition catalyst. The scale bar represents 20 μm. (b) Intracellular retention of HKYellow. SH-SY5Y cells were stained with HKYellow-AM (10 μM) and then treated with 500 μM SIN-1 for 30 min. After washing three times, the cells were imaged at indicated time points. The scale bar represents 20 μm. (c) Relative mean fluorescence levels of the cells shown in (b) were quantified. Data are the mean ± S.E.M., *n* = 211–246 cells. For each experiment, at least three independent biological replicates (*n* ≥ 3) on different days were performed showing reproducible results.

Next, we sought to apply HKYellow to visualize peroxynitrite in live cells. For efficient loading of the probe into cells, we prepared the acetoxymethyl ester derivative of HKYellow,^35^,^36^ HKYellow-AM (Fig. 1c and Scheme S1 in the ESI†) and used it in all the subsequent imaging experiments. Unlike HKYellow, HKYellow-AM with a caged carboxylate is neutral and can readily diffuse across the cell membrane. Once inside the cells, HKYellow-AM is hydrolyzed by esterases to regenerate HKYellow for reaction with peroxynitrite (Fig. 1c). To further verify the probe selectivity in biological systems, SH-SY5Y human neuroblastoma cells were incubated with HKYellow-AM and then treated with a panel of ROS and RNS donors after brief washing. Cells loaded with HKYellow-AM (10 μM) only showed negligible background fluorescence owing to the non-fluorescent feature of the probe (Fig. 3a). Treatment of the cells with SIN-1, a peroxynitrite generator, triggered a significant increase in the intracellular fluorescence, which could be suppressed by pre-treating the cells with FeTMPyP, a peroxynitrite decomposing catalyst. Moreover, no intracellular fluorescence increase was observed in cells treated with H_2_O_2_, NO donor NOC-18, or O_2_˙^–^ donor MSB (Fig. 3a). Moreover, HKYellow-AM was also applicable in other cell types, such as C17.2 mouse neural progenitor cells, bEnd.3 mouse brain endothelial cells, and primary astrocytes, for the imaging of peroxynitrite (Fig. S7 in the ESI†).

We also evaluated the intracellular retention of HKYellow, which was loaded into the cells through HKYellow-AM. As demonstrated by the strong fluorescence and the limited signal decrease in SH-SY5Y cells over two hours (Fig. 3b and c), HKYellow and the fluorescent product **2** are believed to be well-retained inside the cells, due to the negatively charged carboxylates. Co-staining experiments of HKYellow-AM with ER-Tracker, LysoTracker, MitoTracker, or nuclear stain Hoechst 33342 suggested that the probe is predominantly localized in cytoplasm of cells, without selective subcellular localization (Fig. S8 in the ESI†). In addition, both the intact nuclei indicated by Hoechst 33342 stain and an MTT assay revealed that HKYellow-AM has no cytotoxicity (up to 40 μM) for biological applications (Fig. S9 in the ESI†).

**Fig. 4:**
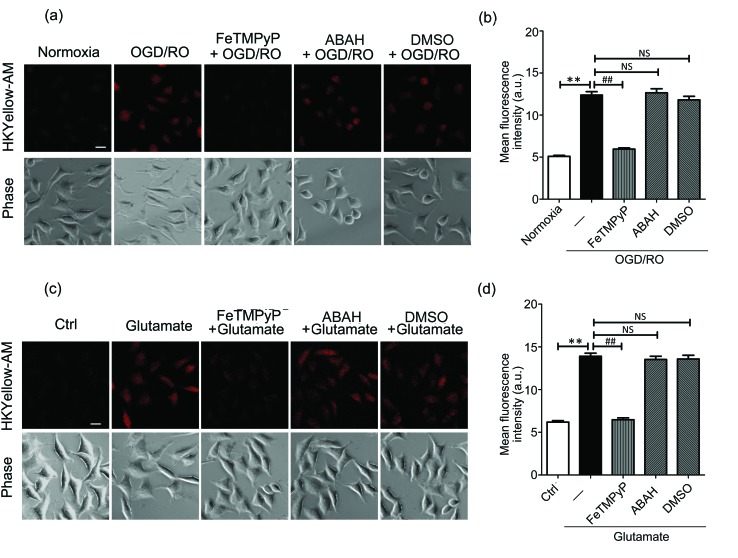
Fluorescence imaging of endogenous peroxynitrite with HKYellow-AM in stimulated SH-SY5Y cells. (a) Cells were subjected to OGD conditions for 10 h, reoxygenation for 1 h and stained with HKYellow-AM (10 μM) before fluorescence imaging. FeTMPyP (50 μM), ABAH (100 μM), or DMSO (1%) was added at the onset of reperfusion to reduce the level of peroxynitrite, HOCl or ˙OH, respectively. The scale bars represent 20 μm. (b) Quantification of the mean fluorescence levels of cells shown in (a). Statistical analyses were performed with one-way ANOVA. Data are the mean ± S.E.M, *n* = 135–238 cells; **p < 0.01 *vs.* normoxia group; ##*p* < 0.01 *vs.* OGD/RO only group; NS, non-significant *vs.* OGD/RO only group. (c) Cells were stimulated with glutamate (5 mM) for 1 h and stained with HKYellow-AM (10 μM) before fluorescence imaging. FeTMPyP (50 μM), ABAH (100 μM), or DMSO (1%) was added to reduce the level of peroxynitrite, HOCl, or ˙OH, respectively. Scale bar represents 20 μm. (d) Quantification of the mean fluorescence levels of cells shown in (c). Statistical analyses were performed with one-way ANOVA. Data are the mean ± S.E.M., *n* = 123–172 cells; **p < 0.01 *vs.* Ctrl group; ##*p* < 0.01 *vs.* glutamate only group; NS, non-significant *vs.* glutamate only group. For each experiment, at least three independent biological replicates (*n* ≥ 3) on different days were performed showing reproducible results.

Then, we sought to visualize the endogenous production of peroxynitrite using HKYellow-AM. It has been known that peroxynitrite is generated during cerebral ischemic injury.^37^,^38^ However, the precise roles of peroxynitrite during this event have been controversial, *e.g.*, whether peroxynitrite is harmful or protective to neuronal cells.^39^ Therefore, direct imaging of peroxynitrite production in the setting of ischemia–reperfusion injury would be crucial and interesting. To mimic ischemia–reperfusion conditions, we utilized an oxygen and glucose deprivation and reoxygenation (OGD/RO) model.^40^ SH-SY5Y cells were maintained with glucose-free DMEM in an anaerobic chamber for oxygen and glucose deprivation, and then returned to normal incubator for reoxygenation, followed by incubation with HKYellow-AM and fluorescence imaging. As shown in Fig. 4a, the cells subjected to OGD/RO conditions exhibited an increased fluorescence when compared to those cultured in normoxia conditions. In addition, the fluorescence increase was attenuated by treating the cells with FeTMPyP, but it was not affected by the myeloperoxidase inhibitor, 4-aminobenzoic acid (ABAH) or ˙OH radical scavenger DMSO (Fig. 4a and b), thus confirming that peroxynitrite was indeed generated under OGD/RO conditions. During ischemia conditions, glutamate is released into the extracellular space as an excitatory neurotransmitter and eventually induces the generation of ROS and RNS.^41^ To examine the involvement of peroxynitrite in glutamate excitotoxicity, SH-SY5Y cells were stimulated with glutamate (5 mM) for 1 h and stained with HKYellow-AM. As shown in Fig. 4c and d, strong fluorescence increases were observed in the glutamate-treated cells when compared to the control and FeTMPyP treated cells. Moreover, treating the cells with ABAH and DMSO did not abrogate the fluorescence increase. Together, these results indicate that HKYellow is capable of detecting endogenous bursts of peroxynitrite produced in neuronal cells under OGD/RO conditions or glutamate stimulation conditions.

### Imaging of peroxynitrite with HKYellow-AM in live tissues

**Fig. 5:**
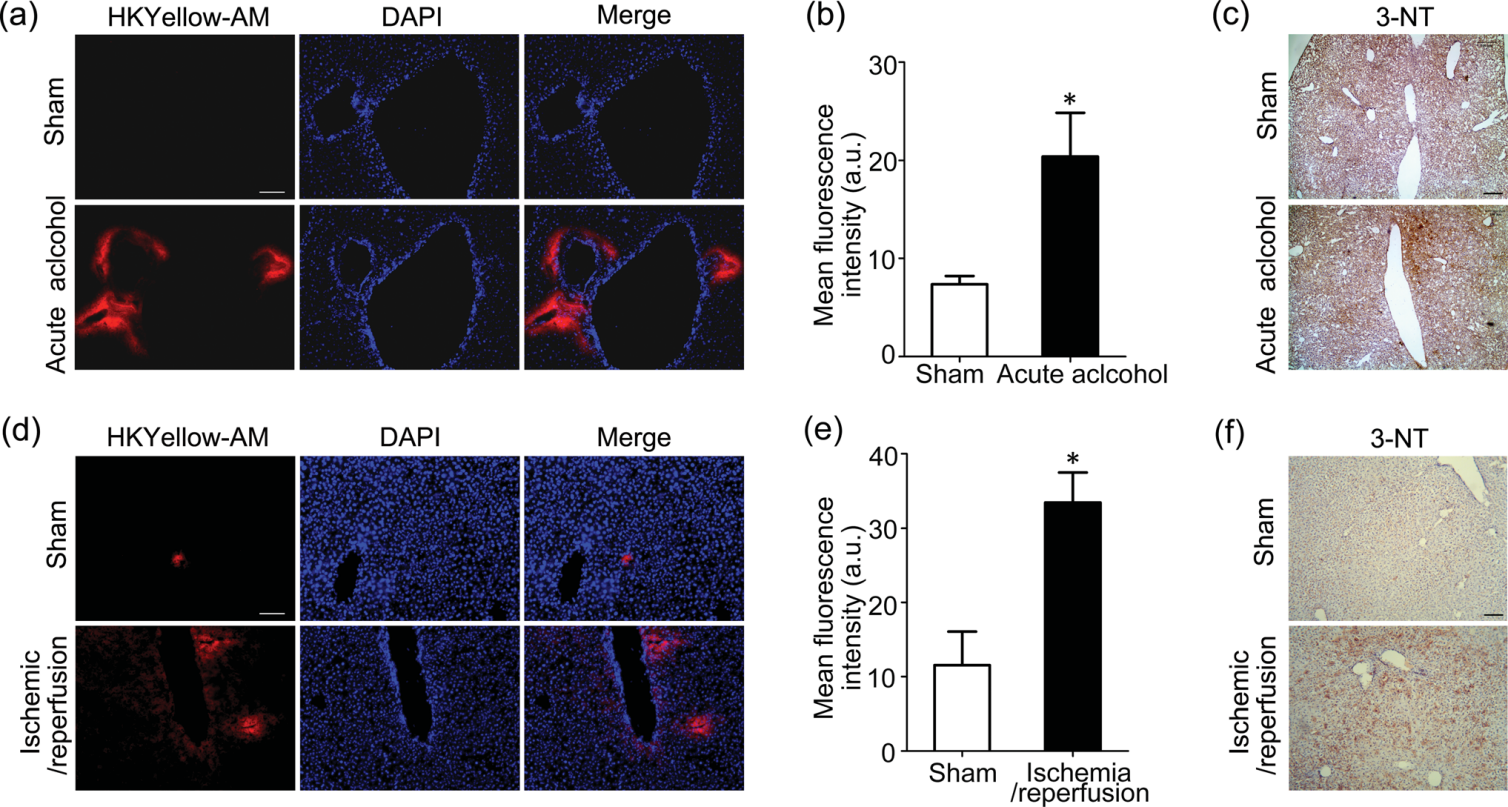
Molecular imaging of endogenous peroxynitrite with HKYellow in mouse livers. (a–c) Mice in the acute alcohol model group were given 50% ethanol, whereas the sham group mice received the same amount of water (*n* = 6 per group). (d–f) For the ischemia/reperfusion mouse model, atraumatic clips were applied to the portal vessels of mouse hepatic lobes for 1 h and then removed. The sham group mice were processed without portal vessel clamping. (a and d) Mouse livers were *in situ* perfused with HKYellow-AM (20 μm; perfusion rate: 2 mL min^–1^, total 25 mL), cryosectioned, and mounted for fluorescence imaging. DAPI was used to stain the nucleus. (b) Quantification of the mean HKYellow fluorescence levels of liver tissue shown in (a). Statistical analyses were performed with the student's *t*-test. Data are shown as the mean ± S.E.M., *p < 0.05 *vs.* sham group. (e) Quantification of the mean HKYellow fluorescence levels of liver tissue shown in (d). Statistical analyses were performed with the student's *t*-test. Data are shown as the mean ± S.E.M., *p < 0.05 *vs.* sham group. (c and f) Mouse livers were cryosectioned and immunostained with 3-nitrotyrosine (3-NT) antibody. The scale bars represent 100 μm.

As our final goal, we would like to evaluate the ability of this unique chemical tool to image peroxynitrite in animal tissues. First, HKYellow was shown to be able to visualize exogenous peroxynitrite in cultured *ex vivo* rat brain slices (Fig. S10 in the ESI†), confirming that the probe is applicable for tissue imaging. We then applied HKYellow-AM to image the generation of endogenous peroxynitrite in live tissues. It has been previously implicated that peroxynitrite may be involved in the progression of acute alcohol-induced liver injury^42^,^43^ and hepatic ischemic/reperfusion injury^44^ on the basis of immunostaining of protein nitration, a footprint reaction of peroxynitrite commonly used for its indirect detection. However, to the best of our knowledge, direct evidence for peroxynitrite generation in these contexts is still missing. To this end, we employed mouse models with acute alcohol-induced liver injury^45^ or hepatic ischemia/reperfusion injury^46^ for imaging endogenous peroxynitrite generation. Briefly, for acute alcohol-induced liver injury model, the alcohol group mice were given 50% ethanol, whereas sham group mice were supplied with water. For the hepatic ischemia/reperfusion injury model, atraumatic clips were applied to the portal vessels of the median and left mouse hepatic lobes for 1 h to mimic ischemia conditions. The clips were then removed to initiate 6 h reperfusion. After respective treatment, the mice were anesthetized and livers were *in situ* perfused with HKYellow-AM. Then, the fresh livers were cryosectioned and imaged. As shown in Fig. 5a–b and d–e, marked fluorescence increases were observed surrounding the vascular vessels in alcohol-treated or ischemic/reperfused mouse liver samples, whereas negligible fluorescence signals were observed in the control liver samples, which is consistent with our imaging studies on a cellular level (Fig. 4). Moreover, an immunostaining assay using antibody against tyrosine nitration consistently suggested the level of protein nitration was indeed remarkably increased in mouse liver samples with either acute alcohol treatment or ischemic/reperfusion induction (Fig. 5c and f). Taken together, these results suggest that HKYellow-AM is compatible with histological sample preparation and can be applied to imaging endogenous peroxynitrite in live tissues. More importantly, our study provides the first direct imaging evidence showing elevated peroxynitrite generation in acute alcohol-induced liver injury and hepatic ischemic/reperfusion injury conditions that may contribute to oxidative damage to biomolecules during liver injury.

## Conclusions

To summarize, we presented the rational development of a robust fluorescent probe HKYellow for molecular imaging of peroxynitrite utilizing a peroxynitrite-induced *N*-dearylation reaction and a unique rhodamine core whose fluorescence intensity is dramatically and selectively turned on by peroxynitrite. HKYellow and its cell loading derivative HKYellow-AM have been thoroughly and stringently evaluated in both chemical and biological systems to exhibit features including favorable excitation/emission wavelengths, high sensitivity and selectivity to peroxynitrite, fast reaction, photostability, broad pH range, superior intracellular retention, no cytotoxicity (up to 40 μM), and broad biological applicability. These features are highly desirable for applying HKYellow to the molecular imaging of peroxynitrite in live cells and tissues. Indeed, taking advantage of this new probe, we directly visualized endogenous peroxynitrite production in live cells under OGD/RO conditions or upon treatment with excitatory glutamate. More importantly, HKYellow has been extensively utilized for molecular imaging of peroxynitrite in live mouse liver tissues, enabling direct visualization of endogenous peroxynitrite generation in mouse livers under acute alcohol binge or ischemic–reperfusion conditions for the first time and therefore providing new insights into the pathological mechanisms of these two important disease conditions. Given its robustness and versatileness, HKYellow should also serve as a powerful molecular imaging tool for studying the biology of peroxynitrite under various physiological and pathological contexts in the future. Moreover, the rational design strategy for HKYellow based on the fluorescence quenching effect of the *N*-phenyl group to xanthene fluorophores and a peroxynitrite-triggered *N*-dearylation reaction can be generalized to other core fluorophores to further expand our toolbox of peroxynitrite probes, which will be reported in due course.

## Supplementary Material

Supplementary information
