# Supplementary material for: A rationally designed rhodamine-based fluorescent probe for molecular imaging of peroxynitrite in live cells and tissues
Source: Chem Sci. 2016 Apr 26;7(8):5407–13. doi: 10.1039/c6sc00012f (PMC6020818; doi:10.1039/c6sc00012f)
Supplement: Supplementary file 1 [file SC-007-C6SC00012F-s001.pdf]

Electronic Supplementary Material (ESI) for Chemical Science.
This journal is © The Royal Society of Chemistry 2016

Supporting Information

A Rationally Designed Rhodamine-based Fluorescent Probe for Molecular Imaging of Peroxynitrite

*Tao Peng, Xingmiao Chen, Lei Gao, Ting Zhang, Wei Wang, Jiangang Shen, and Dan Yang**

1. Synthetic methods and materials

All chemicals were purchased from Aldrich, Acros, or Alfa Aesar, and used as received without further purification. All solvents were used after appropriate distillation or purification. Reactions were performed in oven-dried apparatus under an inert atmosphere (e.g., Ar or N_2) when necessary. Air- and moisture-sensitive reagents were introduced via syringes through rubber septa. Reactions were monitored by thin layer chromatography (TLC) using E. Merck silica gel 60 precoated glass plates with 0.25 mm thickness. Compounds were visualized by illumination with a short-wavelength ultra-violet light and/or staining in phosphomolybdic acid (PMA) or $KMnO_4$ solution followed by heating. Flash column chromatography was performed on E. Merck silica gel 60 (230−400 mesh ASTM).

NMR spectra were recorded in $CDCl_3$ or CD_3OD at ambient temperature on a Bruker Avance DPX 300 Fourier Transform Spectrometer operating at 300 MHz for ^1H and at 75.47 MHz for ^{13}C or Bruker Avance DPX 400 Fourier Transform Spectrometer operating at 400 MHz for ^1H and at 100.6 MHz for ^{13}C. ^1H NMR chemical shifts were reported using tetramethylsilane (TMS, δ 0.00 ppm) or CD_3OD (CD_3, δ 3.31 ppm) as internal standard. ^{13}C NMR chemical shifts were reported using the central peak of $CDCl_3$ (δ 77.00 ppm) or CD_3OD (δ 49.00 ppm) as internal standard.

Mass spectra were recorded with a Finnigan MAT 95 mass spectrometer for both low resolution and high resolution analysis. HPLC analysis was performed with an Agilent 1100 HPLC system. LC-MS analysis was performed with an Agilent 6120 Quadrupole LC/MS System coupled to the HPLC system using ESI and APCI ionization sources.

2. Syntheses and characterization

Scheme S1. Synthetic scheme for HKYellow and HKYellow-AM.

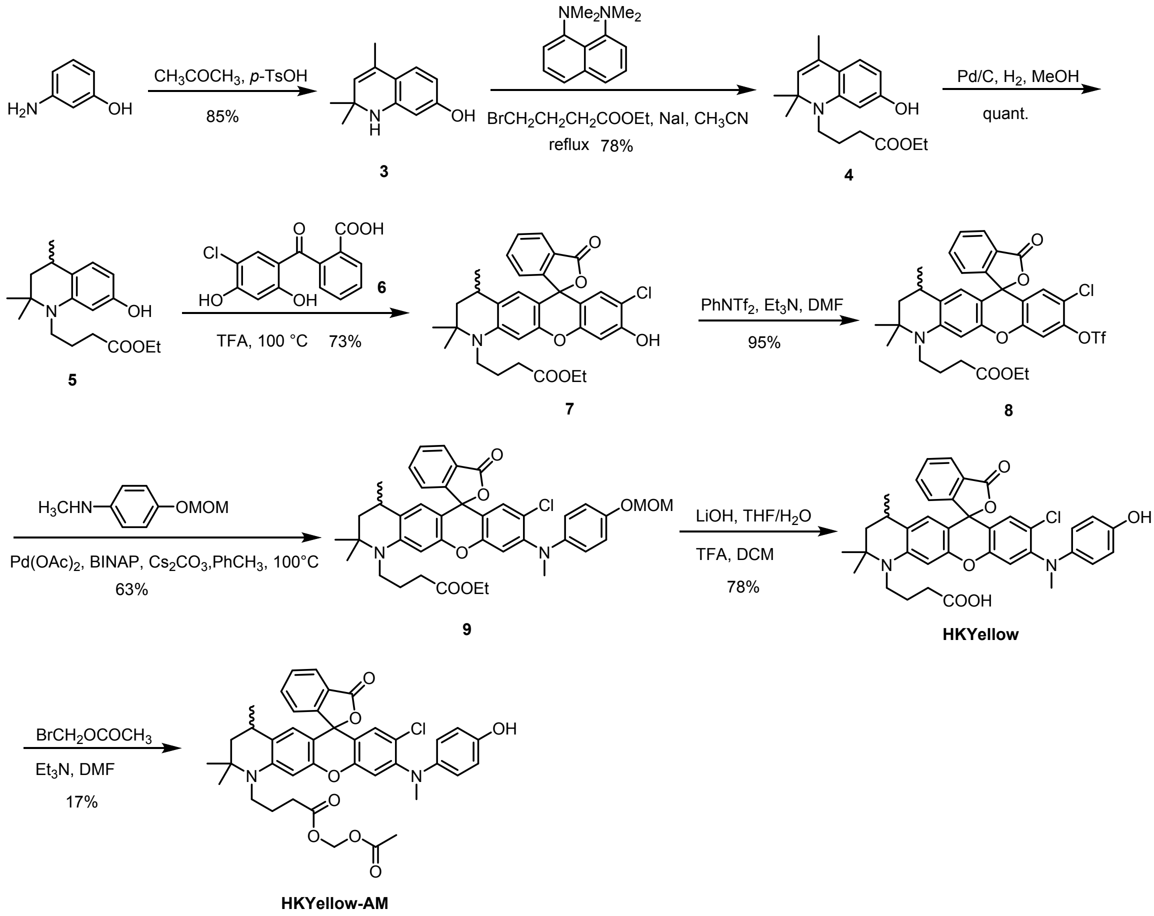

Synthesis of 3. To a solution of 3-aminophenol (10.2 g, 93.6 mmol) in acetone (300 mL) was added *p*-toluenesulfonic acid monohydrate (3.6 g, 18.7 mmol). The mixture was stirred at room temperature overnight, and then concentrated *in vacuo*. The residue was purified by silica gel column chromatography to provide the product **3** (15.0 g, 85% yield). ^{1}H NMR (400 MHz, CD_3OD) δ 6.85 (d, J = 8.1 Hz, 1H), 6.07 – 6.03 (m, 2H), 5.14 (s, 1H), 4.86 (br, 2H), 1.90 (s, 3H), 1.20 (s, 6H); ^{13}C NMR (100 MHz, CD_3OD) δ 158.75, 146.65, 129.56, 126.79, 125.38, 116.11, 105.13, 101.25, 52.40, 30.46, 18.78; LRMS (EI) *m/z* (%): 189 (M^+, 100); HRMS (EI): calcd for $C_{12}H_{15}NO$ (M^+), 189.1154; found, 189.1156.

Synthesis of 4. To a solution of **3** (2.85 g, 15.1 mmol) in anhydrous CH_3CN (40 mL) were added NaI (1.13 g, 7.5 mmol), proton sponge (3.87 g, 18.1 mmol), and ethyl 4-bromobutanoate (2.6 mL, 18.1 mmol) at room temperature under Ar. The resulting suspension was heated to reflux overnight. After cooled to rt, the solid was filtered off with Celite, and the filtrate was concentrated. The residue was re-dissolved in ethyl acetate, washed with dilute HCl, and concentrated. The resulting residue was purified by silica gel column chromatography to give the product **4** (3.5 g, 78% yield). ^{1}H NMR (400 MHz, $CDCl_3$) δ 6.89 (d, J = 7.8 Hz, 1H), 6.39 (br, 1H), 6.12 – 6.10 (m, 2H), 5.07 (s, 1H),

4.16 (q, J = 7.0 Hz, 2H), 3.19 (t, J = 7.7 Hz, 2H), 2.38 (t, J = 6.7 Hz, 2H), 1.92 – 1.90 (m, 5H), 1.29 – 1.26 (m, 9H); ^{13}C NMR (100 MHz, $CDCl_3$) δ 173.98, 156.80, 145.32, 127.56, 126.57, 124.74, 116.29, 102.30, 98.24, 60.75, 56.79, 43.38, 31.63, 28.28, 23.35, 18.71, 14.18; LRMS (EI) *m/z* (%): 303 (M^+, 100); HRMS (EI): calcd for $C_{18}H_{25}NO_3$ (M^+), 303.1834; found, 303.1834.

Synthesis of 5. To a solution of **4** (3.5 g, 11.6 mmol) in degassed MeOH (100 mL) was slowly added palladium (10% on activated carbon powder, 350 mg). The mixture was hydrogenated with a H_2 balloon overnight at room temperature. The mixture was then filtered through a pad of Celite, and the filtrate was concentrated *in vacuo*. The residue was purified by silica gel column chromatography to give compound **5** (two isomers in 1:1 ratio, 3.5 g, ~quantitative yield). ^1H NMR (400 MHz, $CDCl_3$) δ 6.93 (d, J = 8.8 Hz, 1H), 6.38 (br, 1H), 6.18 – 6.11 (m, 2H), 4.16 (q, J = 7.1 Hz, 2H), 3.36 – 3.24 (m, 1H), 3.07 – 2.96 (m, 1H), 2.84 – 2.73 (m, 1H), 2.35 (t, J = 6.9 Hz, 2H), 1.98 – 1.91 (m, 1H), 1.89 – 1.78 (m, 1H), 1.68 (dd, J = 12.9, 4.7 Hz, 1H), 1.48 (t, J = 12.9 Hz, 1H), 1.29 – 1.25 (m, 9H), 1.13 (s, 3H); ^{13}C NMR (100 MHz, $CDCl_3$) δ 174.10, 155.28, 145.95, 126.58, 120.35, 102.35, 98.81, 60.77, 54.49, 47.31, 44.50, 31.70, 29.60, 26.77, 24.91, 24.14, 20.24, 14.25; LRMS (EI) *m/z* (%): 305 (M^+, 54), 290 (100), 204 (91); HRMS (EI): calcd for $C_{18}H_{27}NO_3$ (M^+), 305.1985; found, 305.1988.

Synthesis of 6. The mixture of 2,7-dichlorofluorescein (4 g, 10 mmol) and aqueous NaOH solution (50% w/v, 125 mL) was heated to reflux for 1 hr. After cooled to room temperature, the solution was carefully acidified with concentrated HCl until large amounts of precipitates were formed. The solids were then collected by filtration, and recrystallized with hot MeOH to give the product **6** (2.8 g, 9.6 mmol, 96% yield). ^1H NMR (400 MHz, CD_3OD) δ 8.12 (dd, J = 7.8, 1.3 Hz, 1H), 7.72 (td, J = 7.8, 1.3 Hz, 1H), 7.65 (td, J = 7.8, 1.3 Hz, 1H), 7.38 (dd, J = 7.8, 1.3 Hz, 1H), 6.95 (s, 1H), 6.48 (s, 1H); ^{13}C NMR (100 MHz, CD_3OD) δ 202.28, 168.56, 164.63, 161.67, 141.41, 134.63, 133.65, 131.61, 131.01, 130.55, 128.50, 115.51, 113.20, 104.79; LRMS (EI) *m/z* (%): 293 (M^+, 23), 248 (100); HRMS (EI): calcd for $C_{14}H_9ClO_5$ (M^+), 292.0139; found, 292.0139.

Synthesis of 7. The suspension of **5** (464 mg, 1.5 mmol) and **6** (443 mg, 1.5 mmol) in anhydrous TFA (10 mL) was heated to 100 ºC in a sealed tube under Ar protection for 3 h. After cooled to room temperature, the solution was concentrated and azeotroped with toluene three times to provide the crude product, which was subjected to silica gel column chromatography to give the pure compound **7** (two isomers in 1:1 ratio, 615 mg, 73% yield). ^1H NMR (400 MHz, $CDCl_3$) δ 8.15 (d, J = 6.9 Hz, 1H), 7.68 – 7.61 (m, 2H), 7.22 – 7.18 (m, 1H), 6.86 (s, 1H), 6.79 (s, 0.5 × 1H), 6.77 (s, 0.5 × 1H), 6.56 (s, 1H), 6.48 (s, 1H), 4.22 (br, 1H), 4.20 (q, J = 7.0 Hz, 2H), 3.55 – 3.43 (m, 1H), 3.30 – 3.17 (m, 1H), 2.80 – 2.60 (m, 1H), 2.45 (t, J = 6.5 Hz, 2H), 2.05 – 1.88 (m, 2H), 1.69 (d, J = 12.9 Hz, 1H), 1.51 – 1.47 (m, 1H), 1.35 (s, 3H), 1.31 (t, J = 7.0 Hz, 3H), 1.21 (s, 3H), 1.07 (d, J = 6.3 Hz, 0.5 × 3H), 0.99 (d, J = 6.3 Hz, 0.5 × 3H); ^{13}C NMR (100 MHz, $CDCl_3$; values are given for one isomer with those of the second isomer in brackets) δ 173.21, 169.11 (169.08), 154.14, 153.47, 149.88, 144.46, 133.30, 130.56, 129.79, 128.34, 127.93, 127.59 (127.25), 126.62 (126.53), 125.30, 124.80, 120.99, 113.48, 108.49 (108.38),

104.00, 97.50, 60.85, 56.22 (56.09), 51.91, 45.98 (45.88), 45.10 (44.98), 31.69, 29.54 (29.40), 26.94 (26.79), 25.96 (25.65), 23.45 (23.33), 19.58 (19.40), 14.41; LRMS (EI) *m/z* (%): 562 (M^+, 30), 518 (100); HRMS (EI): calcd for $C_{32}H_{32}ClNO_6$ (M^+), 561.1918; found, 561.1919.

Synthesis of 8. To a solution of **7** (615 mg, 1.1 mmol) in DMF (10 mL) were added Et_3N (0.3 mL, 2.2 mmol) and *N*-phenyl-bis(trifluoromethanesulfonimide) (589 mg, 1.6 mmol) under Ar at room temperature. The mixture was stirred overnight and then diluted with ethyl acetate. The organic solution was washed with HCl solution, water, and dried over anhydrous sodium sulfate, and then concentrated *in vacuo*. The residue was purified by silica gel column chromatography to give the product **8** (723 mg, 95% yield). ^1H NMR (400 MHz, $CDCl_3$) δ 8.06 (d, *J* = 7.2 Hz, 1H), 7.77 – 7.63 (m, 2H), 7.33 (s, 0.5 × 1H), 7.32 (s, 0.5 × 1H), 7.23 (d, *J* = 7.2 Hz, 0.5 × 1H), 7.21 (d, *J* = 7.2 Hz, 0.5 × 1H), 6.90 (s, 1H), 6.44 (s, 0.5 × 1H), 6.43 (s, 0.5 × 1H), 6.40 (s, 1H), 4.22 (t, *J* = 7.1 Hz, 2H), 3.52 – 3.36 (m, 1H), 3.25 – 3.10 (m, 1H), 2.78 – 2.57 (m, 1H), 2.44 (t, *J* = 6.8 Hz, 2H), 2.05 – 1.96 (m, 2H), 1.74 – 1.64 (m, 1H), 1.48 – 1.42 (m, 1H), 1.35 – 1.30 (m, 6H), 1.17 (s, 3H), 1.06 (d, *J* = 6.6 Hz, 0.5 × 3H), 0.96 (d, *J* = 6.6 Hz, 0.5 × 3H); ^{13}C NMR (100 MHz, $CDCl_3$; values are given for one isomer with those of the second isomer in brackets) δ 173.20 (173.18), 169.11 (169.08), 152.27 (152.05), 151.06 (150.99), 150.69 (150.57), 147.57 (147.37), 145.89 (145.85), 135.43 (135.36), 130.28 (130.23), 130.15 (130.11), 126.85 (126.61), 126.54 (126.11), 125.35, 124.68, 124.11 (123.99), 121.43 (121.36), 120.91, 118.67 (q, $J_{C\text{-}F}$ = 318.92 Hz), 112.13, 103.61 (103.60), 97.79 (97.75), 82.73 (82.58), 60.70, 55.19 (55.05), 46.38 (46.29), 44.71 (44.51), 31.67 (31.65), 29.49 (29.36), 26.91 (26.75), 25.74 (25.29), 23.52 (23.36), 19.68 (19.55), 14.34; ^{19}F NMR (376 MHz, $CDCl_3$) δ -73.17; LRMS (EI) *m/z* (%): 694 (M^+, 11), 649 (18), 516 (100); HRMS (EI): calcd for $C_{33}H_{31}ClF_3NO_8S$ (M^+), 693.1406; found, 693.1389.

Synthesis of 9. An oven-dried Schlenk tube charged with $Pd(OAc)_2$ (21 mg, 0.092 mmol), BINAP (86 mg, 0.14 mmol), and Cs_2CO_3 (359 mg, 1.1 mmol) was flushed with Ar gas for 5 min. A solution of **8** (639 mg, 0.92 mmol) and 4-(methoxymethoxy)-*N*-methylaniline[1] (204 mg, 1.2 mmol) in toluene (5 mL) was added, and the resulting mixture was first stirred under Ar at room temperature for 30 min and then at 100 ºC for 20 h. The reaction mixture was allowed to cool to room temperature, diluted with CH_2Cl_2 and filtered through a pad of Celite. The filter cake was washed with CH_2Cl_2. The filtrate was then concentrated and the residue was purified by silica gel column chromatography to give the product **9** (413 mg, 63% yield). ^1H NMR (400 MHz, $CDCl_3$) δ 8.04 (d, *J* = 7.5 Hz, 1H), 7.72 – 7.62 (m, 2H), 7.26 (d, *J* = 7.5 Hz, 1H), 7.11 (s, 1H), 6.94 (d, *J* = 7.7 Hz, 2H), 6.76 (s, 1H), 6.73 (d, *J* = 7.7 Hz, 2H), 6.38 (s, 2H), 5.11 (s, 2H), 4.19 (q, *J* = 6.9 Hz, 2H), 3.47 (s, 3H), 3.45 – 3.35 (m, 1H), 3.24 (s, 3H), 3.20 – 3.10 (m, 1H), 2.78 – 2.58 (m, 1H), 2.40 (t, *J* = 6.5 Hz, 2H), 2.05 – 1.80 (m, 2H), 1.67 (d, *J* = 12.7 Hz, 1H), 1.49 – 1.41 (m, 1H), 1.34 – 1.25 (m, 6H), 1.16 (s, 3H), 1.05 (d, *J* = 6.1 Hz, 1.5 × 3H), 0.95 (d, *J* = 6.1 Hz, 1.5 × 3H); ^{13}C NMR (100 MHz, $CDCl_3$; values are given for one isomer with those of the second isomer in brackets) δ 173.14 (173.11), 169.37, 152.47 (152.31), 151.33 (151.28), 151.22, 151.06 (150.97), 148.22 (148.16), 147.22, 147.00, 143.75, 134.98 (134.92), 129.79 (129.74), 129.66, 127.19 (127.02), 125.76, 125.37,

125.27, 125.05, 124.74, 124.11 (124.00), 118.27 (118.23), 117.24, 116.93 (116.84), 115.05 (115.02), 104.29, 97.76 (97.71), 95.13, 83.93 (83.76), 60.56, 55.85, 55.02 (54.86), 46.45 (46.36), 44.59 (44.36), 40.54 (40.52), 31.61 (31.59), 29.45 (29.30), 26.82 (26.64), 25.66 (25.14), 23.51 (23.32), 19.68 (19.48), 14.26; LRMS (EI) *m/z* (%): 711 (M^+, 34), 667 (100); HRMS (EI): calcd for $C_{41}H_{43}ClN_2O_7$ (M^+), 710.2759; found, 710.2765.

Synthesis of HKYellow. To a solution of **9** (90 mg, 0.13 mmol) in THF/H_2O (3 mL/1 mL) was added LiOH (10 mg, 0.40 mmol). The mixture was stirred at room temperature until TLC showed that the starting material had disappeared. The mixture was then acidified with HCl and extracted with chloroform three times. The residue upon solvent evaporation was re-dissolved in DCM (2 mL) and treated with TFA (2 mL) for 2 h. The solution was concentrated and azeotroped with toluene. The residue was purified with reverse phase silica gel column chromatography to give the product HKYellow (64 mg, 78% yield). ^1H NMR (400 MHz, CD_3OD) δ 8.38 – 8.33 (m, 1H), 7.89 (t, *J* = 7.4 Hz, 1H), 7.83 (t, *J* = 7.4 Hz, 1H), 7.49 (s, 0.5 × 1H), 7.48 (s, 0.5 × 1H), 7.45 (d, *J* = 7.4 Hz, 1H), 7.31 (s, 1H), 7.10 (s, 0.5 × 1H), 7.06 (s, 0.5 × 1H), 7.02 – 6.92 (m, 3H), 6.76 (d, *J* = 8.7 Hz, 2H), 3.89 – 3.77 (m, 1H), 3.74 – 3.62 (m, 1H), 3.53 (s, 0.5 × 3H), 3.52 (s, 0.5 × 3H), 2.97 – 2.85 (m, 1H), 2.60 – 2.56 (m, 2H), 2.10 – 2.00 (m, 2H), 1.96 (dd, *J* = 13.6, 4.0 Hz, 1H), 1.61 (td, *J* = 13.6, 5.0 Hz, 1H), 1.54 (s, 3H), 1.43 (s, 0.5 × 3H), 1.42 (s, 0.5 × 3H), 1.13 (d, *J* = 3.4 Hz, 0.5 × 3H), 1.12 (d, *J* = 3.4 Hz, 0.5 × 3H); ^{13}C NMR (100 MHz, CD_3OD; values are given for one isomer with those of the second isomer in brackets) δ 176.56, 168.26 (168.21), 159.26 (159.17), 157.60 (157.57), 156.92 (156.89), 156.07 (155.93), 155.37 (155.33), 141.29, 135.10, 134.91, 134.78 (134.76), 134.13 (134.10), 132.51 (132.41), 131.88, 131.71, 131.56, 131.33, 126.85 (126.82), 126.73, 125.88, 117.56 (117.49), 117.14, 117.05 (117.00), 107.62 (107.60), 98.46 (98.41), 60.51 (60.48), 47.26, 45.27 (45.25), 44.68 (44.67), 31.33, 29.19 (29.16), 28.03, 25.98, 23.94, 19.07 (18.97); LRMS (EI) *m/z* (%): 639 (M^+, 12), 551 (100); HRMS (FAB): calcd for $C_{37}H_{36}ClN_2O_6$ ($[M+H]^+$), 639.2262; found, 639.2268.

Synthesis of HKYellow-AM. To a solution of HKYellow (34 mg, 0.053 mmol) in anhydrous DMF (2 mL) were added Et_3N (75 μL, 0.53 mmol) and bromomethyl acetate (50 μL, 0.53 mmol) successively under Ar. The resulting solution was stirred overnight under Ar and then diluted with ethyl acetate. The mixture was washed with HCl, water, and brine. The organic solution was dried and concentrated. The residue was purified with silica gel column chromatography to give the product HKYellow-AM (6.4 mg, 17% yield). ^1H NMR (400 MHz, $CDCl_3$) δ 8.04 (d, *J* = 7.5 Hz, 1H), 7.76 – 7.61 (m, 2H), 7.28 – 7.21 (m, 1H), 7.09 (s, 1H), 6.72 – 6.69 (m, 5H), 6.42 – 6.34 (m, 2H), 5.78 (s, 0.5 × 2H), 5.77 (s, 0.5 × 2H), 3.48 – 3.36 (m, 1H), 3.23 (s, 3H), 3.14 – 3.12 (m, 1H), 2.76 – 2.57 (m, 1H), 2.54 – 2.44 (m, 2H), 2.12 (s, 3H), 1.95 – 1.93 (m, 2H), 1.73 – 1.62 (m, 1H), 1.44 (q, *J* = 12.2 Hz, 1H), 1.28 (s, 0.5 × 3H), 1.27 (s, 0.5 × 3H), 1.16 (s, 3H), 1.03 (d, *J* = 6.6 Hz, 0.5 × 3H), 0.93 (d, *J* = 6.6 Hz, 0.5 × 3H); ^{13}C NMR (75 MHz, $CDCl_3$; values are given for one isomer with those of the second isomer in brackets) δ 172.11 (172.08), 169.89, 152.31, 152.12, 151.36 (151.32), 151.22 (151.11), 148.84, 147.27, 147.04, 142.50, 135.14 (135.06), 129.87, 129.66, 127.30, 127.11, 126.03, 125.47, 125.17, 124.81, 124.22, 124.04, 119.95, 115.87, 113.79, 104.50, 97.84 (97.76), 79.39, 55.16 (54.99),

46.46 (46.35), 44.39 (44.14), 31.25, 29.45 (29.27), 26.85 (26.68), 25.74, 25.18, 23.17 (22.94), 20.80, 19.70 (19.46); LRMS (ESI) *m/z* (%): 711 ($[M+H]^+$, 100); HRMS (FAB): calcd for $C_{40}H_{40}ClN_2O_8$ ($[M+H]^+$), 711.2473; found, 711.2472.

Scheme S2. Synthetic scheme for compound **2**.

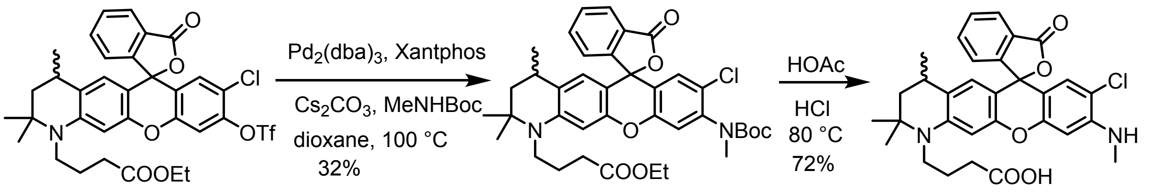

8 **10** **2**

Synthesis of 10. An oven-dried Schlenk tube charged with $Pd_2(dba)_3$ (45 mg, 0.049 mmol), Xantphos (71 mg, 0.12 mmol), and Cs_2CO_3 (88 mg, 0.27 mmol) was flushed with Ar gas for 5 min. A solution of **8** (170 mg, 0.24 mmol) and *tert*-butyl-*N*-methylcarbamate (33 mg, 0.26 mmol) in dioxane (3 mL) was then added, and the resulting mixture was first stirred under Ar at room temperature for 30 min and then at 100 ºC for 36 h. The reaction mixture was allowed to cool to room temperature, diluted with CH_2Cl_2 and filtered through a pad of Celite. The filter cake was washed with CH_2Cl_2. The filtrate was then concentrated and the residue was purified by silica gel column chromatography to give the product **10** (52 mg, 32% yield). ^{1}H NMR (300 MHz, $CDCl_3$) δ 8.05 (d, *J* = 6.8 Hz, 1H), 7.69 – 7.65 (m, 2H), 7.22 – 7.16 (m, 2H), 6.79 (s, 1H), 6.39 (s, 2H), 4.22 (q, *J* = 7.0 Hz, 2H), 3.51 – 3.33 (m, 1H), 3.19 – 3.15 (m, 4H), 2.72 – 2.67 (m, 1H), 2.43 (t, *J* = 6.7 Hz, 2H), 1.98 – 1.93 (m, 2H), 1.66 (d, *J* = 12.0 Hz, 1H), 1.55 – 1.53 (m, 2H), 1.41 – 1.24 (m, 15H), 1.17 (s, 3H), 1.06 (d, *J* = 5.5 Hz, 0.5 × 3H), 0.95 (d, *J* = 5.5 Hz, 0.5 × 3H); LRMS (EI) *m/z* (%): 675 (M^+, 14), 531 (100); HRMS (EI): calcd for $C_{38}H_{43}ClN_2O_7$ (M^+), 674.2759; found, 674.2757.

Synthesis of 2. To a solution of **10** (25 mg, 0.037 mmol) in AcOH (4 mL) was added HCl solution (3 N, 1 mL). The resulting solution was heated to 100 ºC for 1 h and then diluted with water after cooled to room temperature. The mixture was then extracted with DCM/*i*-PrOH three times. The organic layers were combined and concentrated. The residue was purified by silica gel column chromatography to give the product **2** (14.6 mg, 72% yield). ^{1}H NMR (400 MHz, CD_3OD) δ 8.22 (ddd, *J* = 6.3, 5.7, 2.8 Hz, 1H), 7.78 – 7.70 (m, 2H), 7.32 (dd, *J* = 5.4, 2.6 Hz, 1H), 7.18 (s, 1H), 7.16 (s, 1H), 7.05 – 7.02 (m, 1H), 6.92 – 6.90 (m, 1H), 3.79 – 3.71 (m, 1H), 3.64 – 3.54 (m, 1H), 3.06 (s, 3H), 2.92 – 2.79 (m, 1H), 2.57 – 2.47 (m, 2H), 2.01 – 1.98 (m, 2H), 1.96 – 1.85 (m, 1H), 1.64 – 1.53 (m, 1H), 1.50 (s, 3H), 1.37 (s, 3H), 1.14 – 1.10 (m, 3H); ^{13}C NMR (100 MHz, CD_3OD; values are given for one isomer with those of the second isomer in brackets) δ 175.57, 158.65, 158.33, 157.30 (157.23), 156.45 (156.42), 154.94 (154.90), 152.73, 133.33 (133.15), 131.57, 130.27 (130.24), 130.03, 129.37, 128.52, 125.95, 125.69, 119.67, 113.96, 113.47 (113.38), 96.71, 95.12, 78.17, 58.24, 45.56, 44.42, 30.48, 29.23, 28.03, 26.73 (26.70), 24.77 (24.74), 22.76, 17.94 (17.88); LRMS (EI) *m/z* (%): 547 (M^+, 20), 458 (100); HRMS (FAB): calcd for $C_{31}H_{32}ClN_2O_5$ ($[M+H]^+$), 547.2000; found, 547.2005.

3. Photophysical characterization of HKYellow for detection of peroxynitrite

For photophysical characterization, the probes **HKYellow** and **2** were dissolved in DMF or DMSO to make the stock solutions, which were diluted to 2 μM as the testing solutions with phosphate buffer (0.1 M, pH 7.4). UV-visible spectra were recorded on a CARY 50 Bio UV-Visible spectrophotometer. Fluorescence spectroscopic studies were performed on a Hitachi F-7000 fluorescence spectrophotometer. Slit widths were set at 2.5 nm for both excitation and emission spectra, and the photomultiplier voltage was 700 V. To determine the fluorescence quantum yields, rhodamine 6G in ethanol (Φ = 0.95) was used as a reference standard.[1,2]

To test the fluorescence response of the probe **HKYellow** toward various ROS and RNS, aliquots of ROS or RNS solutions were slowly added to the probe solutions (each 5 mL) with vigorously stirring at room temperature in the dark. The volume changes after addition of ROS or RNS solutions were less than 1%. The fluorescence intensities were then measured after 30 min in the dark.

Sources for different ROS/RNS are described as previously.[3] Specifically, $ROO^{\bullet}$ was generated from 2,2'-azobis(2-amidinopropane)dihydrochloride, which was firstly dissolved in deionizer water and then added into the probe solution at 37 °C for 1 h. $^1O_2$ (singlet oxygen) was generated from 3,3'-(naphthalene-1,4-diyl)dipropionic acid. H_2O_2 solution was purchased from Sigma-Aldrich and added into the probe solution directly. The concentration of H_2O_2 solution was determined by iodometric titration prior to use. $^{\bullet}NO$ was generated from sodium nitroferricyanide(III) dihydrate (SNP) and added into probe solution under anaerobic conditions. Briefly, deionized water was degassed with Ar for 20 min. SNP was added into degassed deionized water under Ar atmosphere then the resulting solution was stirred for 30 min at 25 °C. The probe solution was also degassed before the reaction with SNP. Superoxide ($O_2^{\bullet-}$) was generated from xanthine/xanthine oxidase system. Xanthine oxidase was dissolved in the probe solution first, and xanthine in 1.6 M NaOH was added. The mixtures were stirred at 25 °C for 1 h. The source of NaOCl was commercial bleach. The concentration of HOCl was determined by titration with $Na_2S_2O_3$ prior to use. Hydroxyl radical ($^{\bullet}OH$) was generated by Fenton reaction. Briefly, ferrous chloride ($FeCl_2$) was added in the presence of 10 equiv of H_2O_2. The concentration of $^{\bullet}OH$ was equal to the Fe(II) concentration. Peroxynitrite ($ONOO^-$) solution was synthesized according to literature report.[4] Briefly, a mixture of sodium nitrite (0.6 M) and hydrogen peroxide (0.7 M) was acidified with hydrochloric acid (0.6 M), and sodium hydroxide (1.5 M) was added within 1−2 s to make the solution alkaline. The excess hydrogen peroxide was removed by passing the solution through a short column of manganese dioxide. The resulting solution was split into small aliquots and stored at −80 °C. The aliquots were thawed immediately before use, and the concentration of peroxynitrite was determined by measuring the absorption of the solution at 302 nm. The extinction coefficient of peroxynitrite solution in 0.1 M NaOH is 1,670 M^{-1} cm^{-1} at 302 nm.

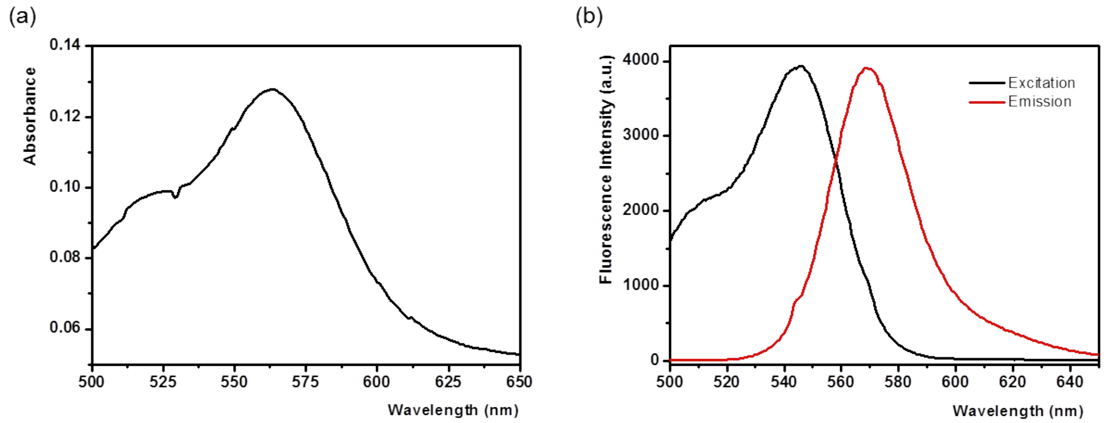

Figure S1. (a) UV-Visible absorption spectrum of **HKYellow** (2 μM) in 0.1 M phosphate buffer at pH 7.4. (b) Fluorescence excitation and emission spectra of compound **2** (2 μM) in 0.1 M phosphate buffer at pH 7.4.

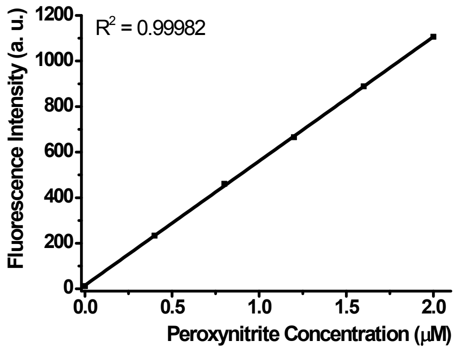

Figure S2. Linear correlation between the fluorescence emission intensity of **HKYellow** (2 μM in 0.1 M phosphate buffer at pH 7.4) and peroxynitrite concentration. The fluorescence intensity was determined at 570 nm with excitation at 545 nm.

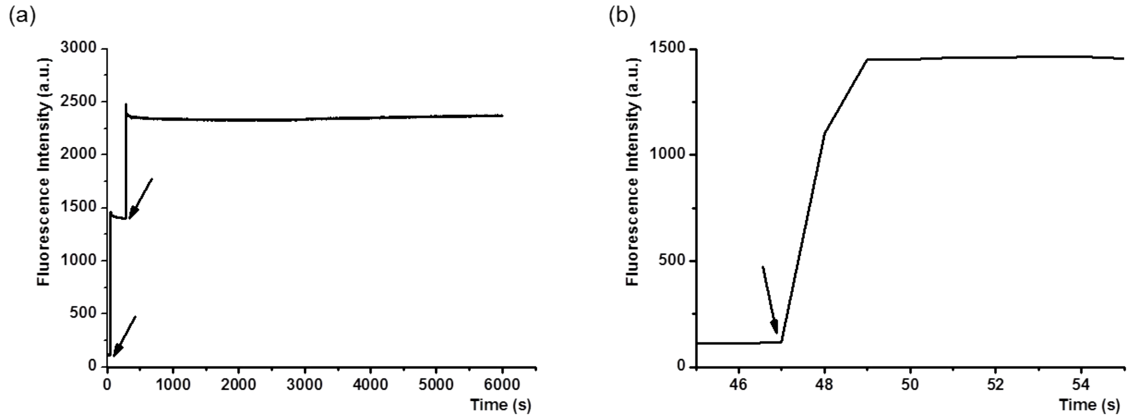

Figure S3. Time course in the detection of peroxynitrite with **HKYellow** monitored by fluorescence. The probe was dissolved in 0.1 M phosphate buffer (pH 7.4) at 2 μM

concentration. The fluorescence intensity was monitored with time at emission wavelength of 570 nm (excitation at 545 nm). Peroxynitrite (1.0 equiv) was added into the probe solution at the time points indicated by the arrows. (a) The fluorescent product of **HKYellow** and peroxynitrite was photostable over 1.5 h. (b) The reaction between **HKYellow** and peroxynitrite was complete within less than 5 seconds.

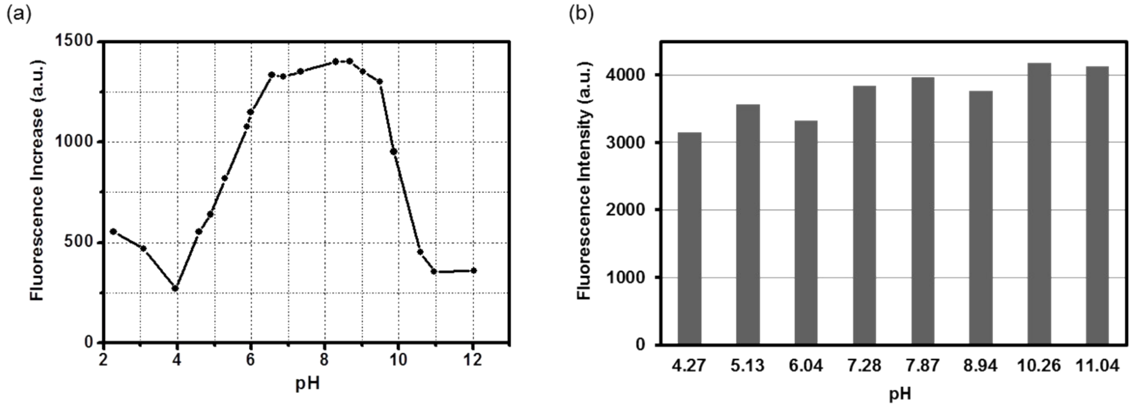

Figure S4. (a) pH-Fluorescence profile of **HKYellow** in the detection of peroxynitrite. The probe was dissolved in pH 7.4 phosphate buffer (0.1 M) at 2 μM concentration. 6 M KOH solution or concentrated phosphoric acid was used to adjust the pH. The final concentration of added peroxynitrite was 2 μM. The fluorescence intensity was recorded at 570 nm with the excitation at 545 nm. The pH differences before and after peroxynitrite addition were determined to be less than 0.05. The left part of this bell-shaped curve is likely ascribed to the acid-base equilibrium of peroxynitrite, while the right part of the bell-shaped curve is probably ascribed to the deprotonation of phenolic hydroxyl group of **HKYellow**. (b) Fluorescence profile of compound **2** at different pH in phosphate buffer. The compound was dissolved in pH 7.4 phosphate buffer (0.1 M) at 2 μM concentration. 6 M KOH solution or concentrated phosphoric acid was used to adjust the pH. The fluorescence intensity was recorded at 570 nm with the excitation at 545 nm.

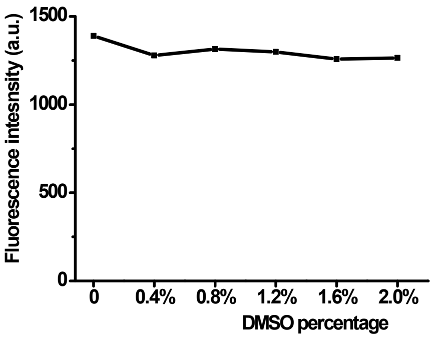

Figure S5. Effects of DMSO in the detection of peroxynitrite with **HKYellow**. Peroxynitrite (1.0 equiv) was added into **HKYellow** solution (2 μM in 0.1 M phosphate buffer at pH 7.4) in the absence or presence of different amounts of DMSO at room

temperature with vigorous stirring. After 30 min, the fluorescence intensity was recorded at emission wavelength of 570 nm (excitation at 545 nm).

4. Reaction of HKYellow with peroxynitrite

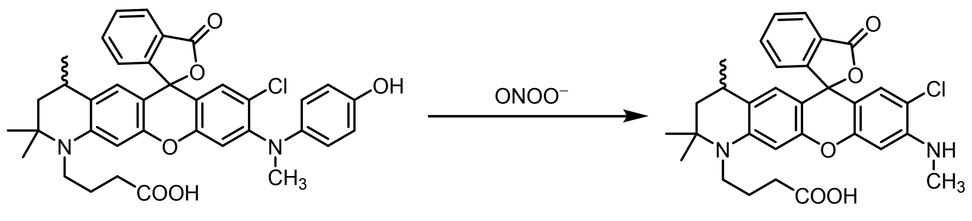

HKYellow **2**

To a solution of probe **HKYellow** (2 μM) in 0.1 M phosphate buffer at pH 7.4 (50 mL) was added an alkaline solution of peroxynitrite (1.0 equiv) dropwise at rt with rigorous stirring. After further stirred for half an hour at rt, the reaction mixture was extracted with DCM/*i*-PrOH three times. The combined organic layers were dried over anhydrous Na_2SO_4 and concentrated. The resulting residue was directly analyzed by HPLC and LCMS (Figure S6). Analytical HPLC was performed with an Agilent 1100 HPLC system. The UV detector was set at 254 nm and 500 nm. The samples were prepared as MeOH stock solutions, and were eluted from an Alltima reverse-phase C18 column (4.6 × 250 mm, 5 μm) with a linear gradient of water (containing 0.1 % TFA) and methanol (60 – 90% methanol in 10 min) at a flow rate of 1 mL/min. Samples were detected by absorbance at 500 nm, and were also identified with an Agilent 6120 Quadrupole LCMS System using ESI and APCI ionization sources.

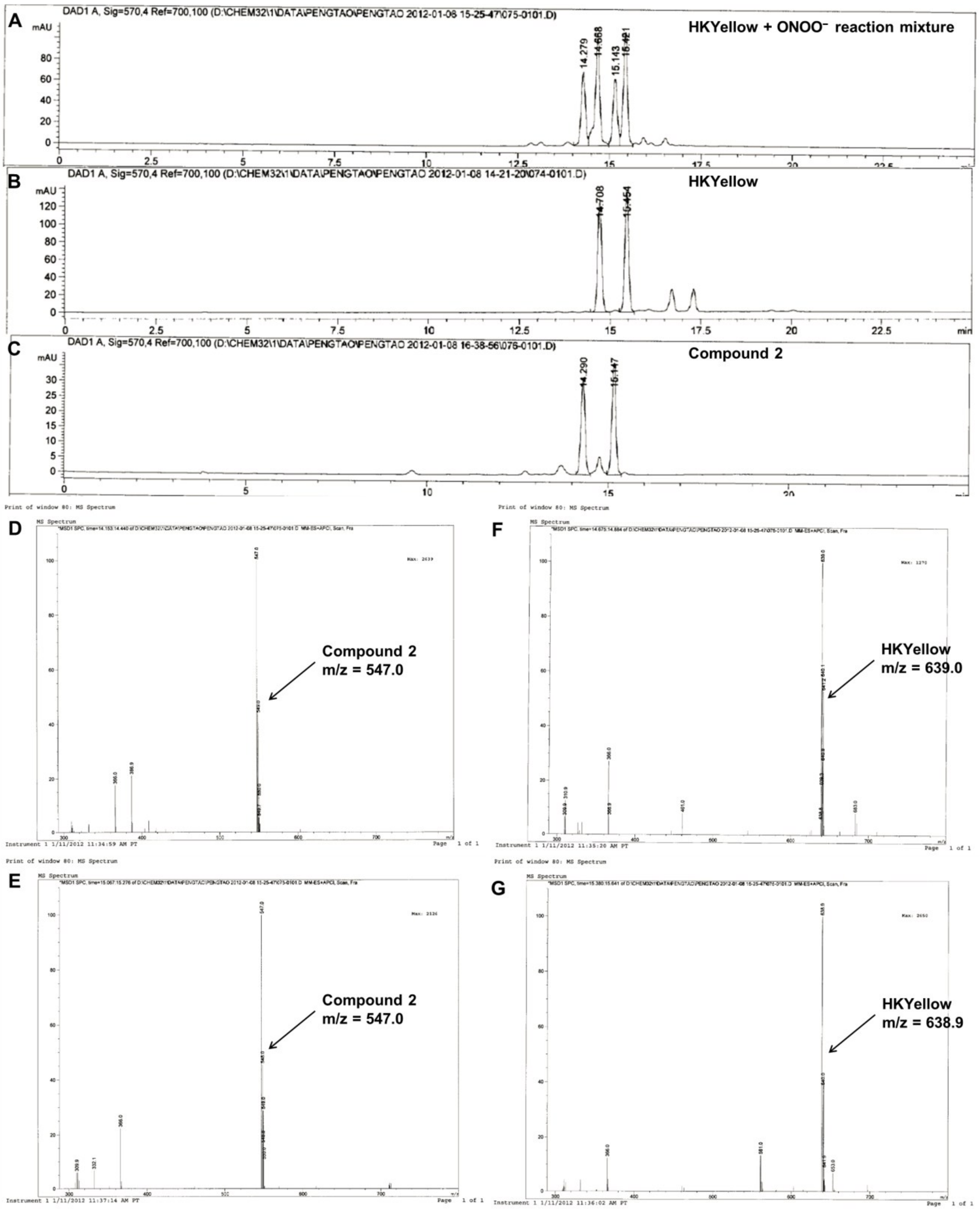

Figure S6. Detection of compound **2** as the fluorescent product in the reaction of **HKYellow** with peroxynitrite using HPLC and LC-MS. UV absorption was monitored at the wavelength of 570 nm with the reference set at 700 nm. (A) HPLC analysis of reaction mixture of **HKYellow** and peroxynitrite with four peaks at retention time of 14.3, 14.7, 15.1, and 15.4 min. (B) HPLC analysis of **HKYellow** with two peaks (two isomers) at retention time of 14.7 and 15.5 min. (C) HPLC analysis of compound **2** with two peaks (two isomers) at retention time of 14.3 and 15.1 min. (D, E, F, and G) MS spectra of

peaks shown in (A) with retention time of (D) 14.3, (E) 15.1, (F) 14.7, and (G) 15.4 min indicate the generation of compound **2** in the reaction of HKYellow with peroxynitrite.

5. Biological Assays of HKYellow-AM

Cell culture

Human neuroblastoma cells SH-SY5Y and bEnd.3 mouse brain endothelial cells were obtained from the American Type Culture Collection (ATCC). Mouse C17.2 neural progenitor cells were a gift from Prof. Godfery Chan in Department of Paediatrics & Adolescent Medicine at The University of Hong Kong. Rat primary cortical astrocytes were prepared from fetal Sprague–Dawley rats (embryonic day 17-18) as previously described.[5] Pregnant rats were obtained from the Laboratory Animal Unit at The University of Hong Kong. Generally, cells were cultured in high glucose Dulbecco's Modified Eagle Medium (DMEM, Hyclone) supplemented with 10% fetal bovine serum (FBS, Life technologies), 1% penicillin/streptomycin (PS, Gibco) and 2 mM L-glutamine (Gibco) at 37 °C with 5% CO_2. Rat primary astrocytes were maintained in neurobasal medium supplemented with 2% B27 (Life technologies) and 0.5% GlutaMAX (Life technologies).

Specificity and localization of HKYellow-AM

SH-SY5Y cells (1×10^5 cells/mL) were seeded onto 12-well plates (Jet Biofil). After overnight culture at 37˚C, cells were washed once with DMEM, and then pre-incubated with 10 μM **HKYellow-AM** for 30 min. After washing with fresh medium for three times, cells were subjected to different treatments for 1 h, including NO donor (1 mM NOC-18, Sigma-Aldrich), superoxide donor (MSB 100 μM, Sigma-Aldrich), H_2O_2 (200 μM), peroxynitrite donor (SIN-1, 100 μM, Cayman Chemical) and the combination of peroxynitrite decomposition catalyst FeTMPyP (50 μM) with SIN-1 (100 μM). After 1 h incubation, cells were subject to fluorescence imaging by a fluorescence microscope (Carl Zeiss, Axio Observer. Z1) equipped with Axio Vision digital imaging system.

For confocal imaging of the intracellular distribution of **HKYellow-AM**, SH-SY5Y cells were seeded onto 35 mm cover-slip dishes (MatTek No. P35G-1.5-10-C). After overnight culture at 37˚C, cells were washed once with DMEM, and then pre-incubated with **HKYellow-AM** (10 μM) for 30 min. After washing with fresh DMEM medium, cells were then treated with SIN-1 (50 μM) for 1 h, and further stained with ER-Tracker Blue, Lyso-Tracker Green, Mito-Tracker Green, and Hoechst 33342 (Life technologies) for selective co-staining of ER, lyososome, mitochondria, and nucleus of live cells, respectively. Cells were then subjected to fluorescence imaging by Multiphoton Confocal Microscope Zeiss 510-Meta (Carl Zeiss) equipped with a live cell detecting system. Confocal imaging was conducted with an oil lens by acquiring 6 consecutive photosections (1 μm per section) at 63x magnification with the following parameters: **HKYellow-AM** (λ_{ex} = 543 nm; λ_{em} = 560-600 nm band-pass).

Intracellular retention of HKYellow-AM

For the retention study, epifluoresence microscopy was used. SH-SY5Y cells (1×10^5 cells/mL) were seeded onto 12-well plates (Jet Biofil). After overnight culture at 37˚C, cells were washed once with DMEM, pre-stained with **HKYellow-AM** (10 μM) for 30 min, and then treated with 500 μM SIN-1 for 30 min before washed with fresh DMEM medium for three times. Cells were imaged at 5 min, 1 h, and 2 h after washing with a fluorescence microscope (Carl Zeiss, Axio Observer. Z1) equipped with Axio Vision digital imaging system. The fluorescent intensity of each image was quantified with Image J (Wayne Rasband, National Institutes of Health, http://rsbweb.nih.gov/ij/).

MTT Assay

3-(4,5-Dimethylthiazol-2-yl)-2,5-diphenyltetrazolium bromide (MTT) assay was utilized to investigate the cytotoxicity of **HKYellow-AM**. Briefly, cells were seeded at a density of 5×10^4 cell per well into a 96-well plate and incubated with 200 μL of culture media overnight. Then different amounts of probe were added to the wells for further incubation with indicated time periods. MTT solution was added to cells at the final concentration of 0.5 mg/ml and incubated with cells at 37°C for 4 h. After removing the culture medium, 150 μl DMSO was added into each well. After continuous shaking for 15 min, the absorbance at 495 nm was measured by Multi-plate Reader (Model 680, Bio-Rad). The cell viability was calculated according to the following equation:

Cell viability (%) = $100 \times A_{\text{with probe}} / A_{\text{control}}$.

Imaging of endogenous peroxynitrite generation in live cells

To mimic the ischemia-reperfusion injury *in vitro*, SH-SY5Y cells were subjected to oxygen glucose deprivation and reoxygenation (OGD/RO) conditions. Briefly, the standard culture medium was replaced with glucose-free DMEM. Cells were then placed in an anaerobic chamber flushed with 95% N_2 and 5% CO_2 and maintained at 37°C for OGD. The concentration of O_2 was monitored with PA-10A paramagnetic O_2 analyzer (Sable Systems International). Following 10 hours of OGD, cells were then removed from the anaerobic chamber, returned to high glucose DMEM, and placed in the standard humidified 5% CO_2 incubator at 37°C for reoxygenation. Meanwhile, **HKYellow-AM** (10 μM) and FeTMPyP (50 μM) were added into the media at the onset of reoxygenaration. After 1 h reoxygenation, images were acquired with a fluorescent microscope (Carl Zeiss, Axio Observer. Z1) equipped with Axio Vision digital imaging system. The fluorescent intensity of each image was quantified with Image J.

To detect the *L*-glutamate induced peroxynitrite generation, SH-SY5Y cells were stimulated with *L*-glutamate (5 mM) for 1 h and stained with **HKYellow-AM** (10 μM). Images were acquired with a fluorescent microscope (Carl Zeiss, Axio Observer. Z1) equipped with Axio Vision digital imaging system. The fluorescent intensity of each image was quantified with Image J.

Imaging of peroxynitrite in ex vivo brain slices

Animal experimental protocol was approved by The University of Hong Kong Institutional Animal Care and Ethical Committee. Sprague–Dawley rats were obtained from the Laboratory Animal Unit at The University of Hong Kong. Briefly, the rats were decapitated and the skulls were quickly opened. After removal of the frontal and occipital poles (including the cerebellum), the isolated brain was immediately placed into ice-cold ACSF (Artificial cerebrospinal fluid) saturated with oxygen. After dissection of the rat brain, the specimens were placed into ACSF (saturated with 95% O_2 to 5% CO_2) and sliced in 300 μm thick sections on a NVSL/NVSLM1 tissue slicer (World Precision Instruments Inc., USA). Slices were collected and maintained in 6-well culture dishes with 1 mL culture medium consisting of 50% minimum essential medium, 24% horse serum, 25% HBSS, and 1% penicillin-streptomycin (all from Invitrogen) and supplemented with 36 mM glucose and 25 mM HEPES (pH 7.2, Sigma). After one day culture, the medium was replaced with fresh medium containing no antibiotics. After 5 days, slices were pre-stained with **HKYellow-AM** (10 μM) for 30 min and then washed with new medium. Slices were then treated with or without SIN-1 (200 μM) and FeTMPyP (50 μM), a catalyst for peroxynitrite decomposition, before monitored by LSM Meta 510 (Carl Zeiss) confocal fluorescence microscope.

Imaging of endogenous peroxynitrite generation in live tissues

Animal experimental protocol was approved by The University of Hong Kong Institutional Animal Care and Ethical Committee. Briefly, 8-12 weeks male mice were used in this study.

For binge drinking model, after 6 hours of fasting, the ethanol group mice were given 50% (vol/vol) ethanol at a total accumulative dosage of 5 g/kg body weight by intragastric administration. After 3 h, mice were anesthetized and then *in situ* perfused with **HKYellow-AM** (20 μM, perfusion rate: 2 mL/min, total 25 mL).

For ischemic/reperfusion model, atraumatic clips were applied to the portal vessels to the median and left hepatic lobes. After 1 hour of ischemia, the clip was removed to initiate hepatic reperfusion. Sham-operated animals underwent laparotomy and liver manipulation without portal vein clamping. After 6h reperfusion, mice were anesthetized and then *in situ* perfused with **HKYellow-AM** (20 μM, perfusion rate: 2 mL/min, total 25 mL). Fresh liver samples were sectioned into 15 μM cryosection slices. After washed with PBS for 5 min and then incubated with DAPI for 10 min, the sections were mounted onto a glass slide (Thermo Scientific) and monitored with a fluorescent microscope (Carl Zeiss, Axio Observer. Z1) equipped with Axio Vision digital imaging system.

Quantification of fluorescence images

For quantification in live cells, we used ImageJ's ROI Manager to select each cell using freeform drawing tools on bright-field images so that the whole cell can be selected. Then we detected the fluorescence intensity of these ROIs in fluorescence images. The mean of the fluorescence intensity of each cell was recorded as Rm.

Additionally, we detected the background intensity of each fluorescence image by randomly selecting three non-cell areas. The average mean of these three random areas was recorded as Bm. Finally we calculated the mean fluorescence intensity of each cell by (Rm - Bm).

For quantification in brain slice images or tissue images, we firstly measured the mean intensity (Rm) and total area (A) of each image with Image J. Then we randomly selected 5 non-tissue areas to calculate the average fluorescence intensity of the background (Bm). Thirdly, we detected all the non-tissue areas in bright-field images and calculated the whole non-tissue areas in each image (B). Finally we used the following equation to estimate the mean fluorescence intensity of each tissue slice by [(Rm-Bm)*A]/(A-B).

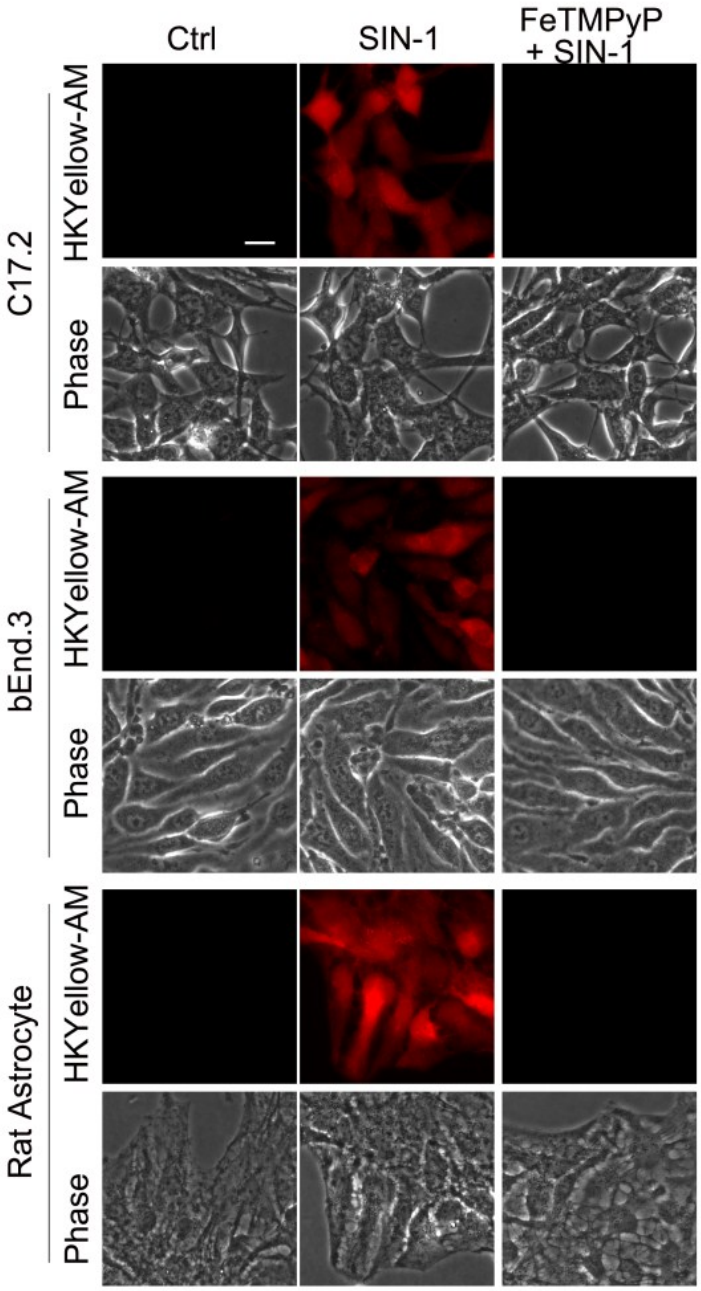

Figure S7. Imaging of exogenous $ONOO^-$ with **HKYellow-AM** in different types of live cells. C17.2 mouse neural progenitor cells, bEnd.3 mouse brain endothelial cells, or primary rat astrocytes were pre-incubated with **HKYellow-AM** (10 μM) for 30 min and then treated with SIN-1 (100 μM) as $ONOO^-$ donor for 1 h, followed by fluorescence imaging. FeTMPyP (50 μM) was used as an $ONOO^-$ decomposition catalyst. Scale bar represents 20 μm.

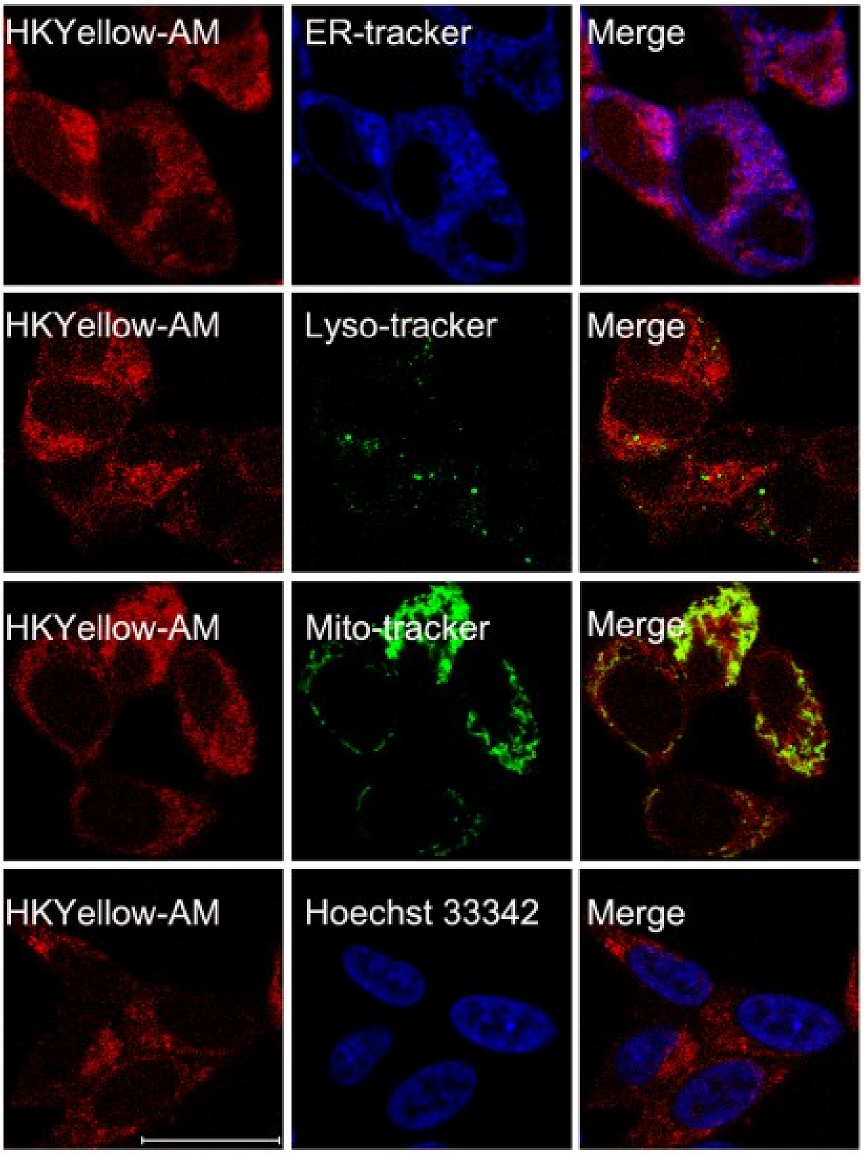

Figure S8. Imaging of **HKYellow-AM** intracellular localization in neuroblastoma SH-SY5Y cells. Cells were pre-incubated with **HKYellow-AM** (10 μM) for 30 mins and then treated with SIN-1 (50 μM) as $ONOO^-$ donor for 1 h, and then stained with ER-Tracker Blue, Lyso-Tracker Green, Mito-Tracker Green, or Hoechst 33342, followed by confocal fluorescence imaging. Scale bar represents 20 μm. HKYellow was excited at 543 nm and LP 560 nm filter was used to collect fluorescence emission. For Mito-tracker-Green and Lyso-tracker-Green, 488 nm laser and BP 500-550 nm filter were used for excitation and emission, respectively. For ER-tracker and Hoechst 33342, 790 nm laser and BP 435-485 nm filter were used for excitation and emission, respectively.

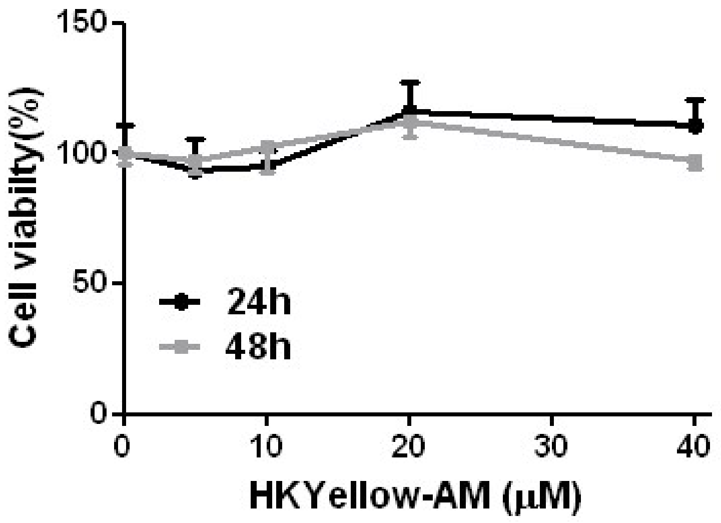

Figure S9. Cytotoxicity of **HKYellow-AM** in neuroblastoma SH-SY5Y cells. Cells were incubated with the probe at corresponding concentrations for 24 or 48 h. Cell viability was measured by MTT assay and the results are reported as percentage relative to untreated cells (mean ± SD).

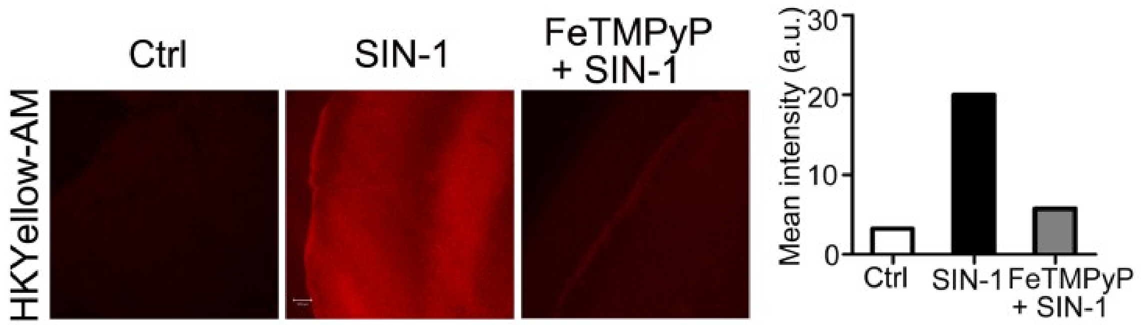

Figure S10. Fluorescence imaging of peroxynitrite with **HKYellow-AM** in *ex vivo* rat brain slices. Rat brain specimens were sliced in 300 μm thick sections and cultured for 5 days before experiments. For staining, brain slices were pre-incubated with **HKYellow-AM** (10 μM) for 30 min and then treated with SIN-1 (200 μM) in the presence or absence of FeTMPyP (50 μM) before imaged by fluorescence microscopy. Scale bar represents 100 μm.

6. NMR spectra

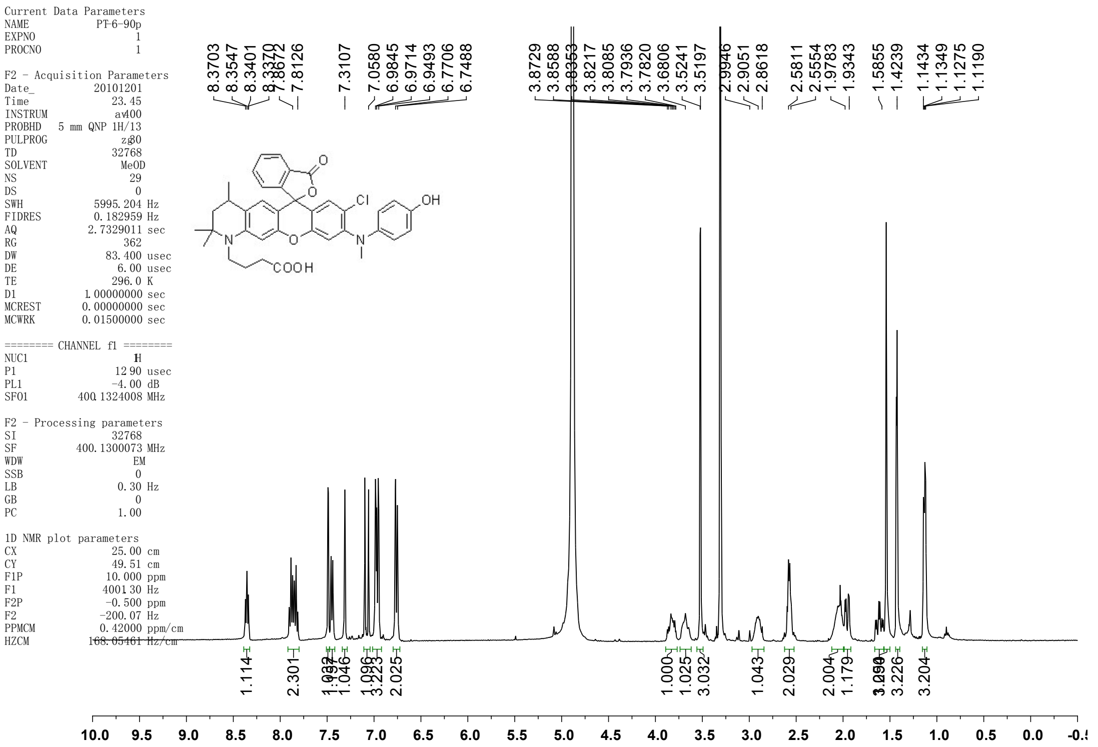

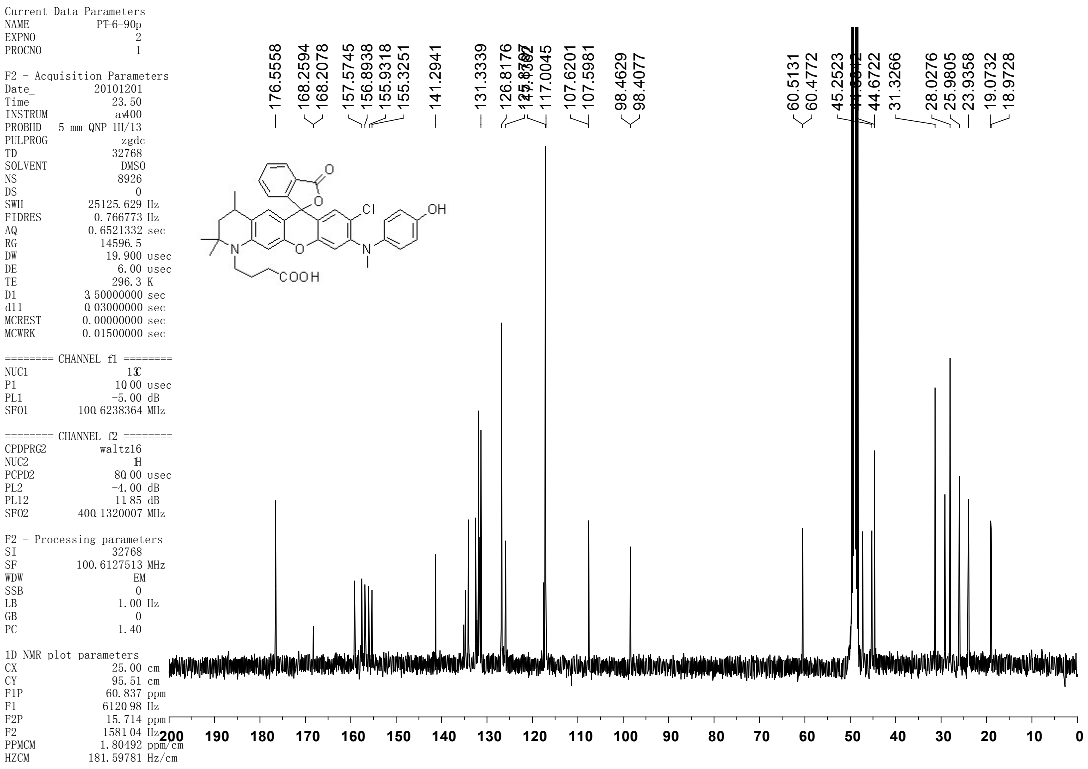

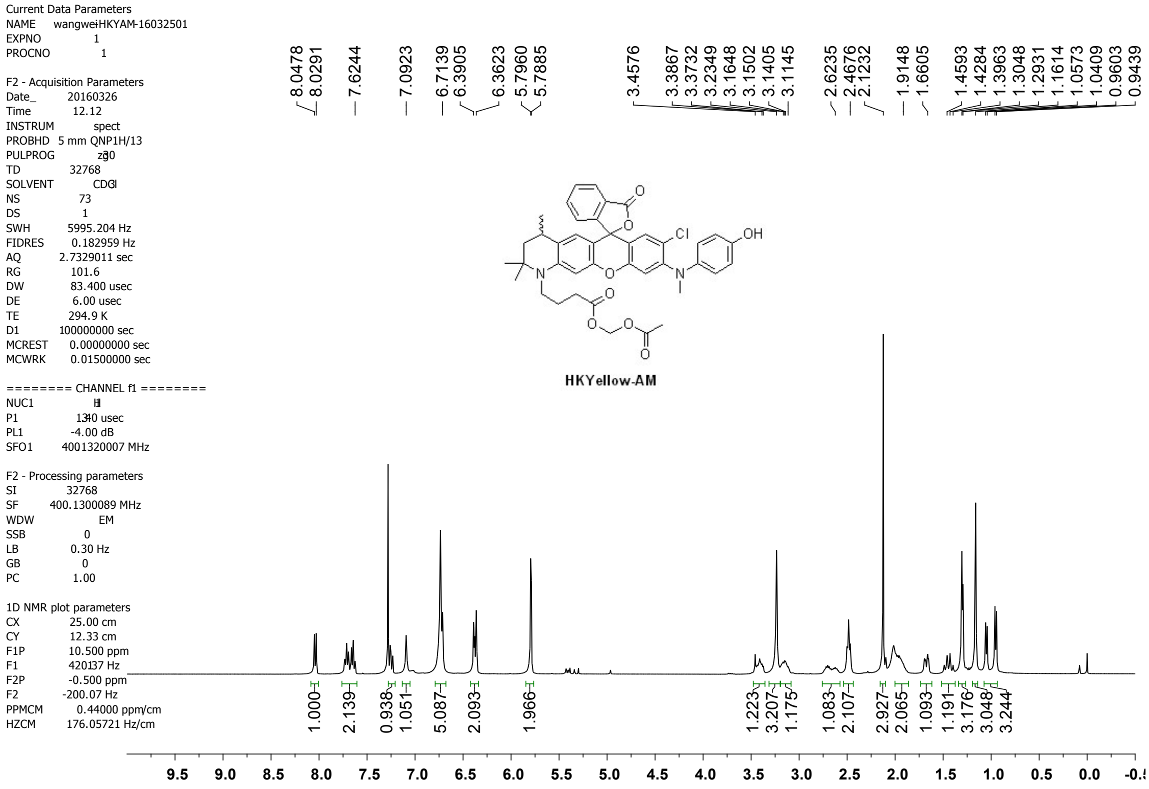

Current Data Parameters
NAME wangweiHKYAM-16032501
EXPNO 1
PROCNO 1
F2 - Acquisition Parameters
Date_ 20160326
Time 12.12
INSTRUM spect
PROBHD 5 mm QNP1H/13
PULPROG zg30
TD 32768
SOLVENT CDCl
NS 73
DS 1
SWH 5995.204 Hz
FIDRES 0.182959 Hz
AQ 2.7329011 sec
RG 101.6
DW 83.400 usec
DE 6.00 usec
TE 294.9 K
D1 100000000 sec
MCREST 0.00000000 sec
MCWRK 0.01500000 sec
======== CHANNEL f1 ========
NUC1 1H
P1 1340 usec
PL1 -4.00 dB
SFO1 4001320007 MHz
F2 - Processing parameters
SI 32768
SF 400.1300089 MHz
WDW EM
SSB 0
LB 0.30 Hz
GB 0
PC 1.00
1D NMR plot parameters
CX 25.00 cm
CY 12.33 cm
F1P 10.500 ppm
F1 420137 Hz
F2P -0.500 ppm
F2 -200.07 Hz
PPMCM 0.44000 ppm/cm
HZCM 176.05721 Hz/cm
8.0478 8.0291 7.6244 7.0923 6.7139 6.3905 6.3623 5.7960 5.7885
3.4576 3.3867 3.3732 3.2349 3.1648 3.1502 3.1405 3.1145 2.6235 2.4676 2.1232 1.9148 1.6605
1.4593 1.4284 1.3963 1.3048 1.2931 1.1614 1.0573 1.0409 0.9603 0.9439
Cl
OH
N
O
HKYellow-AM
1.000 2.139 0.938 1.051 5.087 2.093 1.966 1.223 3.207 1.175 1.083 2.107 2.927 2.065 1.093 1.191 3.176 3.048 3.244
9.5 9.0 8.5 8.0 7.5 7.0 6.5 6.0 5.5 5.0 4.5 4.0 3.5 3.0 2.5 2.0 1.5 1.0 0.5 0.0

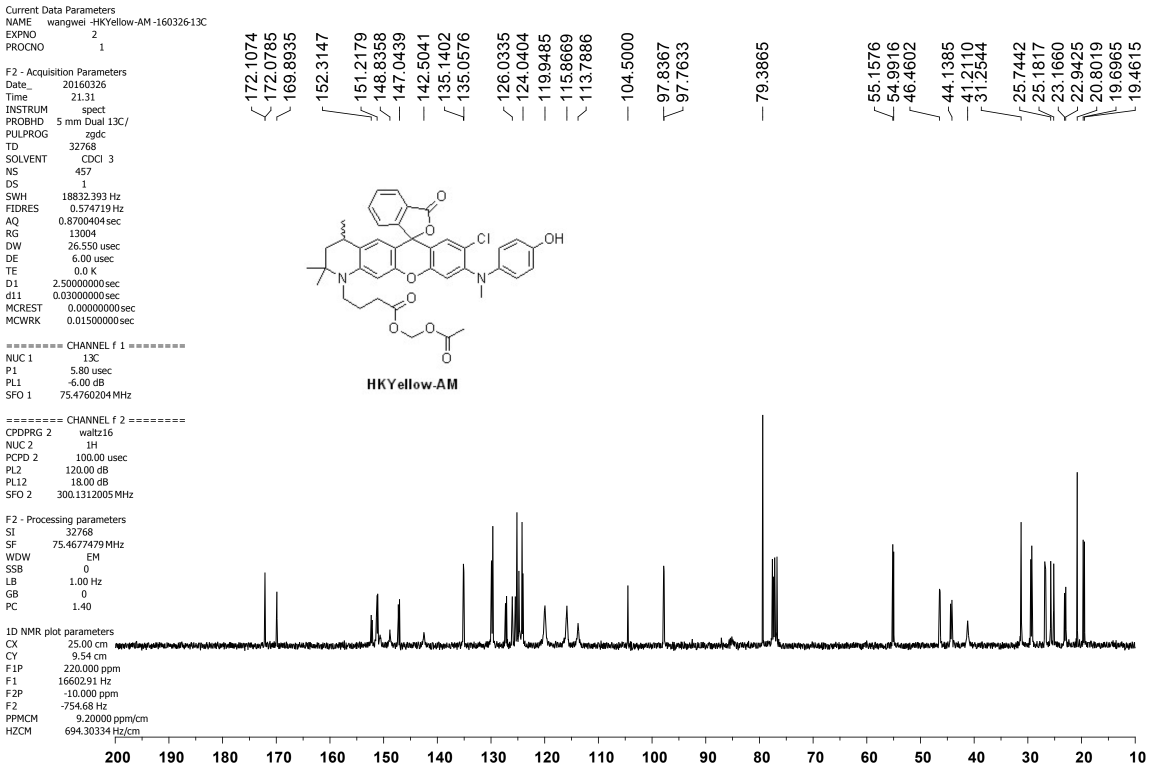

Current Data Parameters
NAME wangwei -HKYellow-AM -160326-13C
EXPNO 2
PROCNO 1
F2 - Acquisition Parameters
Date_ 20160326
Time 21.31
INSTRUM spect
PROBHD 5 mm Dual 13C/
PULPROG zgdc
TD 32768
SOLVENT CDCl 3
NS 457
DS 1
SWH 18832.393 Hz
FIDRES 0.574719 Hz
AQ 0.8700404 sec
RG 13004
DW 26.550 usec
DE 6.00 usec
TE 0.0 K
D1 2.50000000 sec
d11 0.03000000 sec
MCREST 0.00000000 sec
MCWRK 0.01500000 sec
======== CHANNEL f 1 ========
NUC 1 13C
P1 5.80 usec
PL1 -6.00 dB
SFO 1 75.4760204 MHz
======== CHANNEL f 2 ========
CPDPRG 2 waltz16
NUC 2 1H
PCPD 2 100.00 usec
PL2 120.00 dB
PL12 18.00 dB
SFO 2 300.1312005 MHz
F2 - Processing parameters
SI 32768
SF 75.4677479 MHz
WDW EM
SSB 0
LB 1.00 Hz
GB 0
PC 1.40
1D NMR plot parameters
CX 25.00 cm
CY 9.54 cm
F1P 220.000 ppm
F1 16602.91 Hz
F2P -10.000 ppm
F2 -754.68 Hz
PPMCM 9.20000 ppm/cm
HZCM 694.30334 Hz/cm
172.1074 172.0785 169.8935 152.3147 151.2179 148.8358 147.0439 142.5041 135.1402 135.0576 126.0335 124.0404 119.9485 115.8669 113.7886 104.5000 97.8367 97.7633 79.3865
55.1576 54.9916 46.4602 44.1385 41.2110 31.2544 25.7442 25.1817 23.1660 22.9425 20.8019 19.6965 19.4615
Cl
OH
N
O
HKYellow-AM
200 190 180 170 160 150 140 130 120 110 100 90 80 70 60 50 40 30 20 10

7. References

1. T. Peng, N.-K. Wong, X. Chen, Y.-K. Chan, D. H.-H. Ho, Z. Sun, J. J. Hu, J. Shen, H. El-Nezami and D. Yang, *J. Am. Chem. Soc.*, 2014, **136**, 11728-11734.
2. A. M. Brouwer, *Pure Appl. Chem.*, 2011, **83**, 2213-2228.
3. Z.-N. Sun, H.-L. Wang, F.-Q. Liu, Y. Chen, P. K. H. Tam and D. Yang, *Org. Lett.*, 2009, **11**, 1887-1890.
4. J. W. Reed, H. H. Ho and W. L. Jolly, *J. Am. Chem. Soc.*, 1974, **96**, 1248-1249.
5. J. Y. Wang, S. I. Chi, J. Y. Wang, C. P. Hwang and J. Y. Wang, *Chin. J. Physiol.*, 1996, **39**, 227-233.
